# TARGETgene: A Tool for Identification of Potential Therapeutic Targets in Cancer

**DOI:** 10.1371/journal.pone.0043305

**Published:** 2012-08-31

**Authors:** Chia-Chin Wu, David D'Argenio, Shahab Asgharzadeh, Timothy Triche

**Affiliations:** 1 Department of Genomic Medicine, The University of Texas MD Anderson Cancer Center, Houston, Texas, United States of America; 2 Department of Biomedical Engineering and Biomedical Simulations Resource, University of Southern California, Los Angeles, California, United States of America; 3 Children's Hospital Los Angeles and Keck School of Medicine, University of Southern California, Los Angeles, California, United States of America; University of Georgia, United States of America

## Abstract

The vast array of in silico resources and data of high throughput profiling currently available in life sciences research offer the possibility of aiding cancer gene and drug discovery process. Here we propose to take advantage of these resources to develop a tool, TARGETgene, for efficiently identifying mutation drivers, possible therapeutic targets, and drug candidates in cancer. The simple graphical user interface enables rapid, intuitive mapping and analysis at the systems level. Users can find, select, and explore identified target genes and compounds of interest (e.g., novel cancer genes and their enriched biological processes), and validate predictions using user-defined benchmark genes (e.g., target genes detected in RNAi screens) and curated cancer genes via TARGETgene. The high-level capabilities of TARGETgene are also demonstrated through two applications in this paper. The predictions in these two applications were then satisfactorily validated by several ways, including known cancer genes, results of RNAi screens, gene function annotations, and target genes of drugs that have been used or in clinical trial in cancer treatments. TARGETgene is freely available from the Biomedical Simulations Resource web site (http://bmsr.usc.edu/Software/TARGET/TARGET.html).

## Introduction

Intensive use of cytotoxic agents in multimodal therapies has improved five-year disease-free survival and even resulted in cure for some cancer patients. This success can be associated with severe toxicities and an increased occurrence of secondary cancers. The emergence of targeted therapies directed against dysregulated or mutated genes/proteins in malignant cells represents a paradigm shift in cancer therapy, with less reliance on drugs that kill normal cells as well as tumor cells. Examples include therapies against HER2 overexpressed breast cancers (such as Trastuzumab and Lapatinib), c-Kit-targeted therapy in BCR-ABL defective leukemias (Gleevec), and VEGF/VEGF-R-targeted compounds for inhibiting cancerous angiogenesis (such as Bevacizumab). While high throughput technologies such as microarray and next generation sequencing can now be used to identify hundreds or thousands of candidate genes that are differentially expressed or mutated in cancerous versus normal tissues, it is difficult to prioritize potential cancer therapeutic targets from such a large number of candidate genes.

A systematic studying of the complex regulatory pathways is required to understand the mechanisms of oncognesis to discover mutation drivers or develop effective therapies. Several pathways have been found to be deregulated in cancer cells due to the over-expression or repression of some control elements [1]. But, the findings of pathways to date have been very limited. The vast array of high-throughput techniques and public domain data resources more recently available, offers the possibility of understanding cellular mechanisms at a systems level and thus aiding in drug discovery [2]–[5]. Several rigorous statistical approaches have been developed to infer cellular and molecular networks via an integrated analysis of these resources [6]–[10]. We have also previously introduced a Relevance Vector Machine (RVM)-based ensemble approach, designed for large-scale learning problems, and used it to integrate multiple heterogeneous data sources to construct a human gene network that can reveal gene-gene functional relationships [11]. The RVM-based ensemble model yields improved performance on large-scale learning problems with massive missing values in comparison to Naïve Bayes, the most popular method used to predict protein-protein interactions and genetic interactions [6]–[10].

Several concepts also have led to the development of network-based approaches to predict novel disease genes in molecular networks [12], [13], [14]. Genes associated with similar disease phenotypes tend to be group together in a molecular network. Thus, genes that are found to be associated with known disease related genes in the networks are themselves more likely to be involved in the same disease process [12]. In addition, in view of the complexity in cancers, potential therapeutic targets can be those genes/proteins that have a critical role in regulating multiple pathways or maintaining those malignant phenotypes [15]. It has been recently reported that cancer-associated genes are more likely to be signaling proteins that act as hubs, actively sending or receiving signals through multiple pathways [16], [17]. Broader use of these concepts and constructed molecular networks would be promoted by the availability of tools that allow easy identification of potential therapeutic targets for specific cancers.

Broader use of such constructed molecular networks and network-based approaches would be promoted by the availability of tools that allow easy identification of potential therapeutic targets for specific cancers. This report thus introduces the software tool TARGETgene that utilizes a constructed gene network that integrates multiple genomic and proteomic data using the RVM-based model [11] to allow users to conveniently identify potential therapeutic targets for a particular cancer. The network contains not only direct molecular interaction information but also broader gene-gene functional relationships. In addition, by integrating drug-target information compiled from recently available public databases, such as DrugBank [18], PharmGKB [19] and the Therapeutic Target Database [20], TARGETgene allows identification of possible drug candidates for cancer treatments. Users can find, select, and save identified target genes & drugs of interest (e.g., selecting novel cancer genes) via TARGETgene. Through integrating resources from several public databases, TARGETgene also enables users to explore molecular functions, related literature, and enriched biological processes of their selected target genes. Moreover, TARGETgene also provides a way for users to validate their predictions using user-defined benchmark genes (e.g., target genes detected in RNAi screens) and curated cancer genes. In this report, the high-level capabilities of TARGETgene are demonstrated through two applications in this paper: identification of potential therapeutic targets from differentially expressed genes and identification of mutation drivers. The predictions in these two applications were satisfactorily validated in several ways, including known cancer genes, results of RNAi sreens, gene function annotations, and target genes of drugs that have been used or in clinical trials.

## Methods

### Construction of Gene-Gene Functional Relationship Network

Seventeen heterogeneous genomic and proteomic data were integrated using the RVM-based ensemble model reported in [11] in order to construct a gene functional network (as detailed in the section 1 of Text S1). The nodes in this network represent all genes of the human genome, and the functional association between any two of them is quantified by a gene-pair linkage probability that can reveal the tendency of genes to operate in the same or similar pathways. Thus, this network contains not only direct molecular interaction information but also broader functional genetic relationships in pathways. This network can be applied to investigate diverse biological questions in health and disease, including exploring gene functions, understanding complex cellular mechanisms, and identifying potential therapeutic targets. TARGETgene uses this gene network to map and analyze potential therapeutic targets at the systems level.

### Identification of Potential Targets using Network-Based Approaches

Based on the constructed gene network, TARGETgene identifies potential therapeutic targets using one of two network-based metrics: 1) hub score or 2) seed gene association score (as detailed in the section 2 of Text S1). Two centrality measurements, weighted degree centrality and weighted eigenvector centrality, provided in TARGETgene can quantify the tendency of a gene to be a hub in the tumor-specific network that is generated by mapping candidate genes in a tumor to the constructed gene network. TARGETgene also allows users to identify important cancer genes or potential therapeutic targets by associating them with user-defined seed genes (e.g., known cancer genes) in the gene network. More specifically, the importance of each candidate gene is calculated as summation of its direct functional association with those seed genes. All the candidate genes are ranked based on their hub score or their seed gene association score. Those highly ranked genes in the prediction are identified as possible important cancer genes and thus potential therapeutic targets. Drug-target information is then mapped to candidate genes. Drugs whose target genes are highly ranked in the prediction can also be considered as potential therapies.

### Overview of TARGETgene

**Figure 1:**
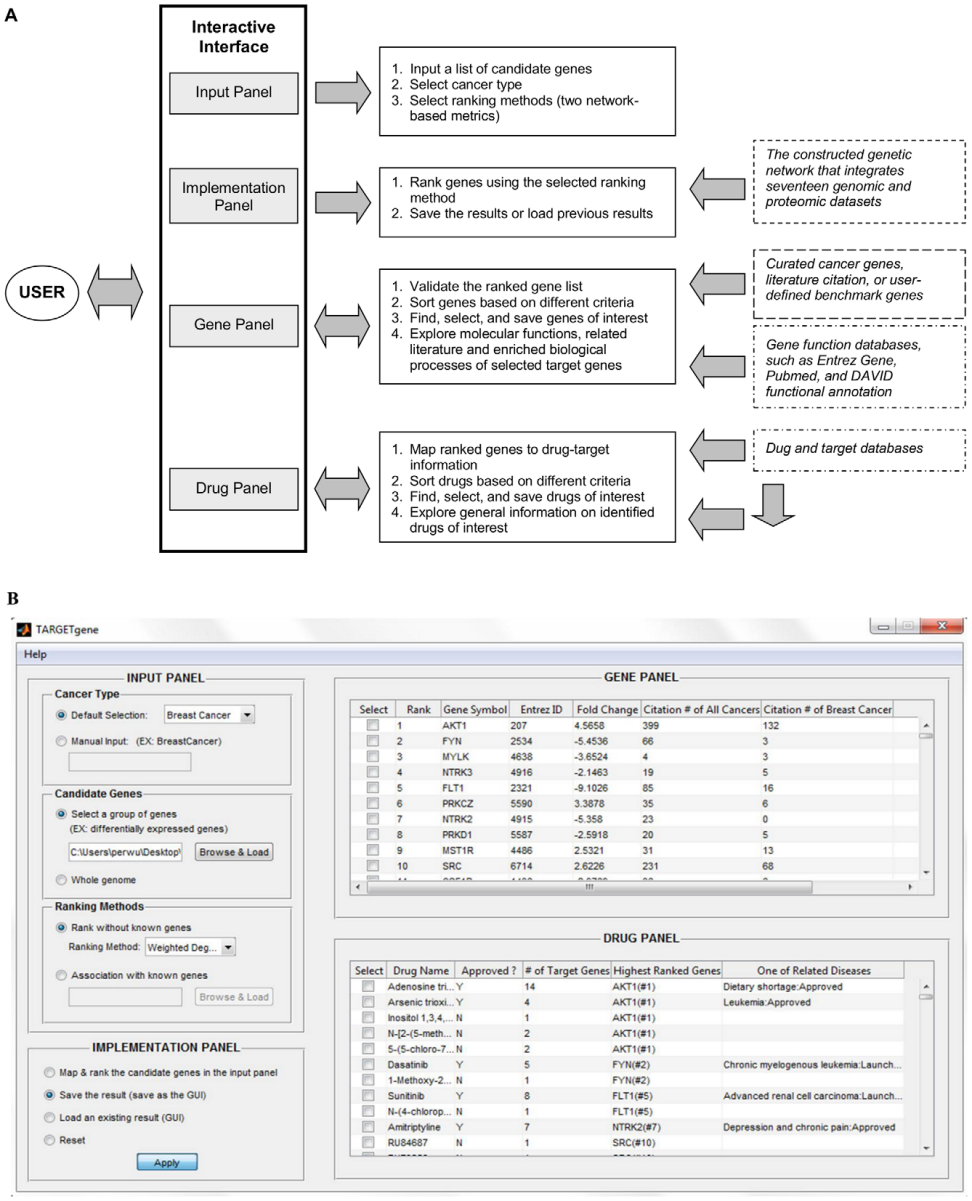
TARGETgene. A. The architecture design. B. The main graphical user interface.

The graphical user interface of TARGETgene consists of four main working panels, including Input, Implementation, Gene, and Drug panels (Figure 1A and 1B). The Input Panel enables users to define the cancer type (currently: breast cancer, colon cancer, Ewing's sarcoma, glioblastoma, lung cancer, ovarian cancer, and prostate cancer) and candidate genes, as well as the desired ranking metric (hub score or seed gene association score) as illustrated in Figure 1A. The Implementation Panel allows the user to generate new predictions, save results and load existing results. The Gene Panel lists information on all candidate genes including their rank in predictions, as well as cancer literature citation number. Cancer literature citation information of genes was compiled from Entrez Gene (ftp://ftp.ncbi.nih.gov/gene/). Through this panel, the user also can find, select identified target genes (e.g., selecting novel cancer genes), and explore their functions, cited literature as well as enriched biological processes. In addition, TARGETgene enables users to validate their predictions using user-defined benchmark genes (e.g., target genes detected in RNAi screens) and curated cancer genes via this panel. Finally, drug and their target information compiled from several public databases, such as DrugBank [18], PharmGKB [19] and the Therapeutic Target Database [20], is also integrated to TARGETgene for reporting those drugs/compounds that could have action on the targets identified by TARGETgene. The Drug Panel lists generic names, drug types (approved or experimental), number of candidate genes known to be targeted by the identified drugs, highest ranked target gene name, and related diseases of the identified drugs. The list of drugs is ordered by their highest ranked target gene. TARGETgene is also customizable and can generate the list of selected drugs based on the ranks of their targets or drug type. In addition, users can explore more general information on identified drugs of interest through several external links.

## Results

To illustrate the use of TARGETgene, we have applied it to two examples: 1) identification of potential therapeutic targets from thousands of differentially expressed genes identified by exon array; 2) identification of driver mutated genes from sequencing and copy number data.

### Example 1: Identification of Potential Therapeutic Targets from Differentially Expressed Genes

In this example, TARGETgene was applied in turn to each of three cancer types: Her2-positive breast cancer, colon cancer, and Lung Adenocarcinoma. Human Exon datasets in the Affymetrix platform for the three cancer types were collected from the National Center for Biotechnology Information Gene Expression Omnibus (GEO) [21]. Subsequent data analyses were done using Partek Genomic Suite 6.3 (more detail in the section 3.1 of Text S1). Finally, 5203, 5153 and 6203 differentially expressed genes were identified in case studies of colon, breast, and lung cancer, respectively. Differentially expressed genes in each cancer type were all ranked based their hub score (weighted degree of centrality) in a tumor-specific network, which was generated by mapping the differentially expressed genes in each cancer type to the constructed gene functional network. The complete ranking list of genes for each of the three cancer types can be obtained by running TARGETgene using the candidate genes list stored in the examples files (included in TARGETgene package) and selecting the weighted degree centrality ranking option (section 3.1 of Text S1 lists the top 10 highest ranked genes for each of the three cancer types as shown in the Gene Panels of TARGETgene). The results show that a number of important cancer genes for each cancer type are ranked highly by TARGETgene, such as AKT1 (rank #1), SRC (rank #10), and ERBB2 (rank #25) in breast cancer. In addition, TARGETgene also ranks several genes highly (in the top 10%) that were recently identified as cancer-related genes in each cancer type. For example, in breast cancer ADAM12 (rank #153) and MAP3K6 (rank #205) were recently reported to be associated with breast cancer oncogenesis [22], [23]. Moreover, many genes that have never been identified in these cancer types are also ranked highly. These genes could be subject to further *in vitro* and *in vivo* study to evaluate their importance these cancer types. Several of these also have been identified by RNAi screens (as detailed in the following section).

#### Prediction Evaluations

The resulting ranked genes from TARGETgene are also validated using gene functional annotations and several benchmark gene sets, including the set of curated cancer genes, the set of genes cited in cancer literature, and the set of target genes detected by RNAi screens. Receiver Operating Characteristic (ROC) Curves and AUC are used for these benchmark evaluations. In each evaluation, the benchmark gene sets are treated as positive instances while others genes are treated as negative instance.

**Figure 2:**
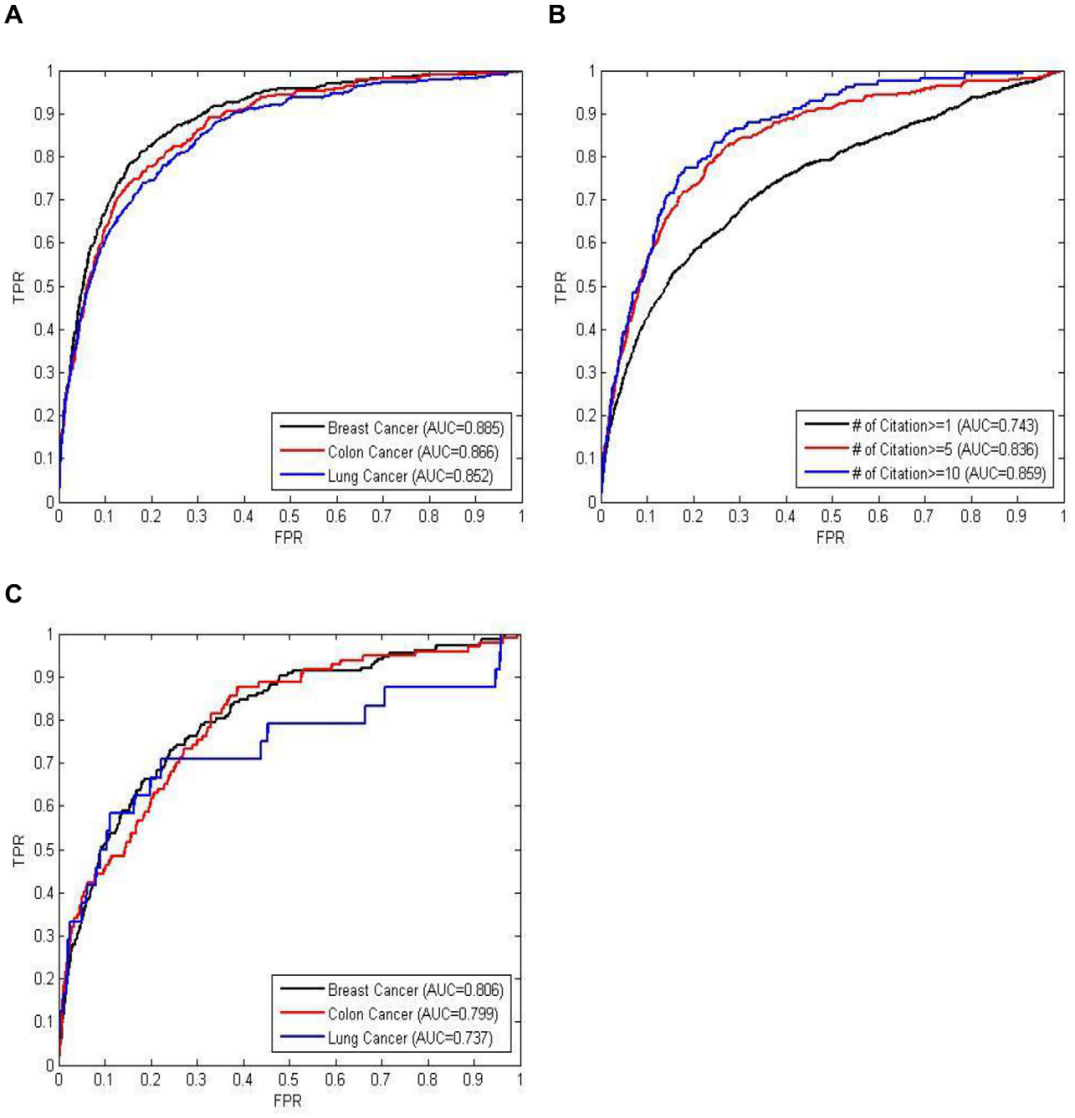
ROC curve performance evaluation for predictions in the example 1. True positive rate is denoted TPR and false positive rate is denoted FPR in the Figure. A. Evaluation using curated cancer genes. B. Evaluation using genes cited by cited by cancer literature with different citation number cutoff values of 1, 5 and 10 (only the case of breast cancer is shown). C. Evaluation using target genes detected by cell viability RNAi screens.

The curated cancer genes downloaded from the CancerGenes database [24] are first used to evaluate if they are highly ranked by TARGETgene. Figure 2A shows TARGETgene's prediction performance for each cancer type. The high AUC values of TARGETgene's prediction in each cancer type (all AUC>0.85) indicate that most of the known cancer genes tend to be ranked highly. In addition, genes that are cited in the literature for each cancer type are also used for evaluation. Benchmark genes in each cancer type can be determined based on different the citation cutoff number. As the citation cutoff number used increases so do the resulting TARGETgene AUC values (Figure 2B shows the result of breast cancer; the results of the other two cases are shown in the section 3.2.1 of Text S1), indicating that genes with more citations also have a higher TARGETgene ranking. The results of Spearman's rank correlation in the three cancer types also shows significant correlation between ranks generated by TARGETgene and literature citation number (section 3.2.1 of Text S1). This provides further evidence that genes highly ranked by TARGETgene are also cited more in the cancer literature; that is, they likely play more important roles in these cancers, compared to lower ranked genes.

High-throughput RNAi screens have recently been shown to be a promising tool to discover new targets for the treatment of several cancers [25]. Therefore, effective targets of each cancer type detected by cell viability RNAi that were downloaded from GenomeRNAi [26] are also applied to evaluate the performance of the predictions from TARGETgene. The data sources of RNAi screens used in this work are summarized in in the section 3.2.3 of Text S1. The result is shown in Figure 2C. The high AUC in each cancer type indicates that the most effected targets identified in the genome-wide RNAi screens tend to be ranked highly by TARGETgene. Some the RNAi target genes that are highly ranked by TARGETgene have been shown to play an important role in oncogenesis in each of the three cancer types, such as AKT1 (#1) in Breast Cancer. Specifically, some of these target genes have only recently been found to be associated with these three cancer types. For example, in breast cancer, PIK3R2 (phosphoinositide-3-kinase, regulatory subunit 2 beta) and ECT2 (epithelial cell transforming sequence 2 oncogene) have a TARGETgene rank of 37 and 272, and with a 3.31 and 4.94 fold change in gene expression of breast cancer tissues, respectively. PIK3R2 has been shown to be functionally associated with unphosphorylated PTEN and the PTEN-associated complex in some HER2-amplified breast cancer cell lines [27]. ECT2 has recently been reported to be involved with mechanisms for activating RhoB after genotoxic stress, thereby facilitating cell death after treatment with DNA damaging agents in Breast Cancer [28]. Most interestingly, we also found that several novel targets (i.e., no citation related to the specific cancer type based on PubMed in Dec. 2010) detected by RNAi screens are also ranked highly by TARGETgene. For examples in Breast Cancer, CASK (calcium/calmodulin-dependent serine protein kinase) and CIT (rho-interacting, serine/threonine kinase 21) are ranked 161 and 115, and with a 2.88 and 3.06 fold change in gene expression of breast cancer tissues, respectively. CASK has been found to be associated with tumorigenesis of esophagus [29]. CIT encodes a serine/threonine-protein kinase that functions in cell division. [30]. Such results provide support on cell line models for the ability of TARGETgene to identify novel therapeutic targets in cancers. This also suggests the possibility of combination of RNAi and network-based screens adopted by TARGTgene for therapeutic target identification (more discussion in the Discussion Section).

Gorilla [31], a gene ontology enrichment analysis tool, was applied to identify enriched GO terms that appear densely at the top of TARGETgene's ranked gene lists for each of the three cancer types. Many of identified GO process terms are known cancer-related biological processes, such as regulation of cell death, regulation of cell proliferation, regulation of cell migration. Interestingly, several biological processes related to new hallmarks of cancers [32] are also identified, such as DNA damage, oxidative stress, evading immune surveillance, metabolic stress, mitotic stress, and proteotoxic stress. These results indicate that genes highly ranked by TARGETgene are involved in multiple cancer-related biological processes and pathways. In addition, several types of molecules, such as signaling kinases, receptor tyrosine kinases, and transcription factors are often proposed as possible molecular targets in cancers [33]–[36]. For example, protein phosphorylation has proven to be an important driving force in cellular signaling [37]. We also find that many kinase, receptor, and transcription factor related GO function terms are enriched in highly-ranked genes in TARGETgene (section 3.2.2 of Text S1). More detail concerning these functional annotations can be found in File S1.

#### Integration of Target Predictions and Drug-Target Information

After mapping the information of drugs/compounds and their targets to the ranked gene lists from TARGETgene, the Drug Panel helps to identify compounds that either have been approved or are currently in clinical trials for the treatment of each of the three cancers. Other drugs and compounds identified by TARGETgene that have not as yet been used in clinical trials, have also shown anti-cancer effect and could thus be considered as potential novel drug for these cancers. Table 1 lists some of these drugs and compounds whose targeted genes are overexpressed and highly ranked by TARGETgene in Breast Cancer (results of Lung and Colon Cancer can be found in the section 3.3 of Text S1). Trastuzumab and Lapatinib have been approved for HER2 positive Breast cancer, and their main target ERRB2 is very highly ranked by TARGETgene (and up-regulated). Several other drugs whose targets are highly ranked by TARGETgene, such as Dasatinib, UCN-01, Celecoxib, Flavopiridol, and Vorinostat, have already been in clinical trials for the treatment of breast cancer. Moreover, other drug/compounds have been shown to have anti-tumor effects and could be considered as potential novel drugs for the treatment in breast cancer, such as Alsterpaullone and Olomoucine. In addition, two naturally occurring compounds, melatonin and vitamin D (Calcidiol), are also identified by TARGETgene. Melatonin, a naturally occurring compound found in organisms, can regulate the circadian rhythms of several biological functions. Recently, a clinical trial involving a total of 643 cancer patients using melatonin found a reduced incidence of death [38]. A study also showed that women with low melatonin levels have an increased risk for breast cancer [39]. Vitamin D receptors have been found in up to 80% of breast cancers, and vitamin D receptor polymorphisms have been associated with differences in survival [40], [41], [42]. Active vitamin D compounds (Calcidiol; Calcitriol) also have been identified for their antiproliferative effects in breast cancer cells [43], [44], although the detail mechanisms are still unclear. In summary, these results provide some further evidence that genes that are highly ranked by TARGETgene can be potential therapeutic targets.

**Table 1:** Selected Drugs Whose Targets Are Highly-Ranked (the case of Breast Cancer).

Drugs/Compounds	Target Genes (Their Ranks and Fold Changes in Cancer)	Literatures Of Breast Cancer Treatment
Dasatinib (E)*	SRC(#10; 2.623)	[45],[46]
Celecoxib (A)*	PDPK1 (#14; 2.917)	[47]
Flavopiridol (E)*	CDK5 (#41; 4.640); CDC2 (#108; 4.382); CDK4 (#50; 2.092)	[48].[49]
Staurosporine(UCN-01) (E)*	PDPK1 (#14; 2.917); MAPKAPK2 (#62; 2.138); CSK (#19; 3.724); GSK3B (#84; 2.130)	[50]
**Alsterpaullone (E)**	CDK5 (#41; 4.640); GSK3B (#84; 2.130); CDC2 (#108; 4.382)	[51]
**Olomoucine (E)**	CDK5 (#41; 4.640); CDC2 (#108; 4.382)	[52]
Trastuzumab (A)***	ERBB2 (#25; 46.856)	[53],[54]
Lapatinib (A)***	ERBB2 (#25; 46.856)	[55],[56]
Dexrazoxane (A)***	TOP2A(#302; 10.965)	[57]
**Lithium (A)**	GSK3B (#84)	[58]
**Melatonin (A)**	CALR(#651)	[59]
**Calcidiol (A)**	VDR (#241)	[44]
Vorinostat (A)*	HDAC3 (#307; 2.336); HDAC1 (#497; 2.286); HDAC2 (#564; 2.520)	[60]
Geldanamycin (17-AAG) (E)*	HSP90B1 (#258; 1.779); HSP90AA1 (#275; 1.920)	[61],[62]
Arsenic trioxide (A)*	AKT1 (#1; 4.566); CCND1 (#418; 3.663)	[63],[64]

**Note**: 1.Approved drugs are denoted as ‘A’.

2.Experimental compounds are denoted as ‘E’.

3.Drugs have been approved for the treatment of Breast Cancer are marked with ***.

4.Drugs in clinical trials for Breast Cancer are marked with *.

### Example 2: Identification of Driver Mutated Genes in Cancer

Large numbers of gene mutations have been discovered from next generation sequencing [65]. A major challenge, however, is to distinguish driver mutated genes that promote the growth of cancer from passenger mutation genes that do not play a role in cancer progression. Several attempts have been made to identified recurrently mutated genes as drivers [66], [67], [68], but thus far these efforts have been unable to detect many drivers unless they are mutated at significantly high frequencies. For example, groups of genes in a pathway that are mutual exclusively mutated. Different combinations of mutations in the same important signaling or regulatory pathway can all generate a significant perturbation and cause cancer development, but these combinations will exclusively appear in a given sample.

**Figure 3:**
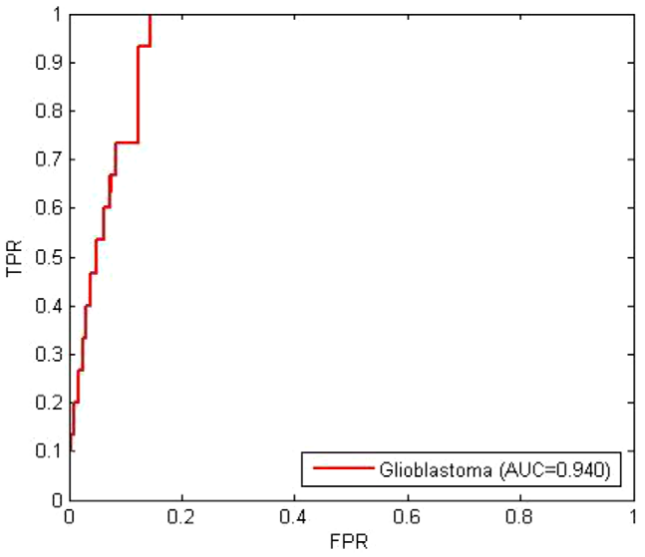
ROC curve performance evaluation for predictions in the example 2. TARGETgene prediction performance is evaluated by genes in the identified core pathways.

Mutations of hub genes in molecular networks are capable of dyregulating the regular functions of many genes and their pathways, due to the ability of hub genes to directly or indirectly alter other components of the cell during their extensive interactions. Accordingly, mutated hub genes may be drivers of cancer progression. In this example, we applied TARGETgene to identify possible driver mutated genes from the approximately 500 mutated genes in the genome of Glioblastoma Multiforme (GBM) [69]. In order to identify those genes whose mutations will have the most significant impacts on other gene, we choose all the genes in the genome as seed genes and then TARGETgene ranked all mutated genes based on their association with all the genes in the genome. One set of genes in the identified core pathways of GBM [68] was used for validation. We found that these genes in the validation core pathways tend to be ranked highly by TARGETgene (AUC = 0.94; Figure 3). Several of these identified core pathway genes are well known GBM genes, such as EGFR (#1), TP53 (#22), and PTEN (#27). It is noteworthy, that two of genes identified by TARGETgene are novel GBM genes (i.e., no GBM literature citations were found), including, CCND2 (#66) and SPRY2 (#68). This indicates highly ranked genes in the TARGETgene prediction may be oncogenic drivers or potential therapeutic targets. In the Drug Panel, TARGETgene also lists some approved drugs that target on those highly ranked genes identified by TARGETgene, some of which have been used for the treatment of GBM or are now in GBM clinical trials (results not shown). All the results can be regenerated by using the Example2 Candidate Genes file on the TARGETgene package and selecting association with all genes in the genome.

## Discussion

### Identification of Potential Therapeutic Targets

Based on the results in the two examples presented, most well studied cancer genes, including those that have shown clinical benefit (e.g., ERBB2 and TOP2 in Table 1), are highly ranked in TARGETgene's predictions in each of the three cancer types. Most notably, TARGETgene also identified several highly ranked genes that are novel in each of the three cancer types. While most new approvals of drugs for target cancer therapies are directed against a few existing targets, such as EGFR, ABL1, only a small number of compounds are in development against novel targets [25]. This indicates that many potential targets remain undiscovered or undrugged. Previous approaches used to identify and validate novel targets in diseases are limited because of high cost, low throughput and time involved [25]. The gene network-based approach as implanted in TARGETgene is able to effectively and comprehensively identify important cancer therapeutic targets. Most importantly, the biological datasets used to construct the gene network are all in the public domains. In addition, although some studies have estimated the size of the “druggable” human genome to be around 10∼20% of human proteome (i.e. the number of the possible protein targets for small-molecule drug design in medicinal chemistry) [70], [71], developing RNAi-based therapies may allow for targeted therapy of virtually any gene [72]. Thus, the targets (up-regulated or mutated) identified by the gene network-based approach in TARGETgene, may all be potential therapeutic targets using RNAi-based therapy. However, most of the targets predicted by TARGETgene still need to be validated in non-clinical models and ultimately in patients.

In the two examples presented, hub genes are identified as important cancer-related genes or potential therapeutic targets using a weighted degree centrality measure. Although the predictions in the three cancer types were satisfactorily validated in several ways, predictions based on this method are expected to be biased toward well-connected genes in the network. For example, some bottleneck hub genes [73] with only a few direct connections to other nodes, but that act as key connectors in the network, may not be identified using the weighted degree centrality measure. The weighted eigenvector centrality measure which can account for the global importance of a gene in the constructed network is an approach for addressing this problem. The constructed gene network used in this study, however, contains not only direct molecular interaction information but also broader (undirected) gene-gene functional relationships, thus reducing the aforementioned selection bias problem when using the weighted degree centrality. We note that comparable prediction performance between the weighted degree centrality and the weighted eigenvector centrality measure, supporting this point (results not shown). However, genes that have not been well-studied to date but may be important in cancer progression will not be identified by the TARGETgene, because little is known about their function. This is a current limitation of TARGETgene for target identification, that may be ameliorated as more genomic and proteomic data are generated and integrated to construct a more complete gene network to be included in future versions of TARGETgene.

### Combination of Predictions of TARGETgene and RNAi Screens

RNAi screens have the ability to identify critical genes that control cancer-related (or disease-related) phenotypes without using any prior biological information. RNAi screens thus can be expected to be a powerful tool for identifying and validating novel targets in the drug discovery process [25]. The gene network-based approach adopted by TARGETgene, however, does not rank some of the targets identified by RNAi screens highly. There are several reasons for the difference between the predicted target using RNAi and the gene network-based approach in TARGETgene. The use of RNAi screens has several significant limitations. First, RNAi screens can only be conducted in cell lines, thus the significance of targets must be further validated in clinical trials. Second, RNAi reagents have off-target effects, which results in the inhibition of genes that are not the intended targets to result in the specific phenotype [25]. Although it is possible to reduce the impact of such effects using extensive validations, only a few targets are finalized and thus generate many false negatives (i.e., many genes that should be targets but are not detected). In contrast, the gene network-based screening in TARGETgene can be used to identify potential therapeutic targets directly using patient data. The approach can also rank all the candidate genes in a cancer based on their functional associations with other genes, and thus may not generate as many false negatives as RNAi screens. An additional advantage of the gene network-based screen is that the pathway information provided in the constructed gene network can be used to interpret the biological processes in which the detected targets are involved, through the inspection of biological roles of related genes. More specifically, the biological roles of groups of functionally related genes of the detected targets can be interpreted by “Gene Enrichment analysis”, which is able to identify major biological processes or pathways associate with these genes. However, it is necessary to imbed prior biological information in the gene network-based approach, which are enriched but still far from complete and may contain some extent of errors.

In principle, RNAi screens could be combined with the gene network-based approach in TARGETgene to arrive at a more refined list of accurate cancer targets without the need for extensive validation of RNAi screens, and with lower false negative rates. The abundant biological information embed in the constructed gene network can provide biological interpretation for the novel targets through their connected genes. In addition, by taking advantage of the gene network-based approach that can identify potential targets using clinical data, one could provide clinical relevance to the novel targets detected by RNAi screens. The gene network-based screen in combination with RNAi screens could be persuasive and provide a complementary mechanism for the identification of therapeutic targets, and thus accelerate drug discovery process.

### Application to Drug Discovery

While the primary purpose of TARGETgene is to identify potential therapeutic targets using integration of heterogeneous biological data, TARGETgene also lists existing drugs and other compounds that may have possible action on the identified targets, as illustrated in the examples presented. These results provide some direct confirmation of abilities of TARGETgene to identify potential drugs. However, these identified drugs may not be effective in the treatment of the indicated cancer for a number of reasons, including: 1). the drug binding affinities are target dependent; 2) the mechanisms of actions of some drugs are unclear; 3). most drugs act against multiple targets, of which some are up-regulated while others are down-regulated in cancers. Therefore, it is difficult to evaluate any possible therapeutic effect of the identified drugs in the predictions.

The results presented in these two applications, however, suggest that TARGETgene could be a tool for initial screening of potential new drugs for further evaluation. Novel drugs whose target genes are highly ranked in TARGETgene's prediction could be considered as potential new drugs for these cancers. These drugs can then be further validated using preclinical testing, such as testing in cell lines or animal models. Most importantly, if targets of some FDA-approved drugs or compounds are highly ranked in the predictions, it is possible to reuse these drugs in the treatment of other cancers or diseases. The results for each cancer type also identify several naturally occurring compounds. Two examples are melatonin and vitamin D whose targets are highly ranked in the case of breast cancer (Table 1).

## Conclusions

There is a vast and diverse amount of public genomic and proteomic resources in the life sciences that may aid in the understanding of disease mechanisms and in the drug discovery process. TARGETgene integrates these resources and provides a platform that enables people to efficiently identify mutation drivers, possible therapeutic targets, and drug candidates in cancer. TARGETgene can rapidly extract gene functional interactions from a precompiled database that is stored as a MATLAB MAT-file without the need to interrogate remote SQL databases. Millions of interactions of thousands of candidate genes can be extracted from the gene network within minutes. While TARGETgene is currently based on the gene network reported in [11], it can be easily extended to allow use of other developed gene networks as options.

One study successfully applied a single gene network to accurately predict tissue-specific phenotypic effects of gene perturbation in Caenorhabditis elegans [9]. In this work, the two examples presented above using TARGETgene further support this possibility. This suggests that the constructed gene network [11] adopted by TARGETgene not only contains critical pathway information, but also can be used to identifying potential therapeutic targets and driver mutations in diverse types of cancer. In addition, existing drugs and other compounds that may have possible action on the identified targets are also provided by TARGETgene. Of course, it is difficult to evaluate any possible therapeutic effect of the identified drugs in the prediction for a number of reasons. However, TARGETgene can be viewed as initial drug screening tool that identifies compounds for be further evaluation. Finally, TARGETgene may also have applications in drug repurposing by identifying compounds that are in use for the treatment of other diseases.

## Supporting Information

File S1 **Enriched GO terms that appear densely at the top of TARGETgene's ranked gene lists for each of the three cancer types.**
(XLS)

Text S1 **Supporting information text.**
(DOC)
