# Supplementary material for: TARGETgene: A Tool for Identification of Potential Therapeutic Targets in Cancer
Source: PLoS One. 2012 Aug 31;7(8):e43305. doi: 10.1371/journal.pone.0043305 (PMC3432038; doi:10.1371/journal.pone.0043305)
Supplement: Text S1 — Supporting information text. (DOC) [file pone.0043305.s002.doc]

**SUPPLEMNTARY MATERIAL**

**TARGETgene: A Tool for Identification of Potential Therapeutic Targets in Cancer**

Chia-Chin Wu^1,*^, David Z. D'Argenio^2^, Shahab Asgharzadeh^3^, Timothy J. Triche^3^

^1^ Department of Genomic Medicine, The University of Texas MD Anderson Cancer Center, Houston, TX, 77030

^2^Department of Biomedical Engineering and Biomedical Simulations Resource, University of Southern, Los Angeles, CA, 90089

^3^Children’s Hospital Los Angeles and Keck School of Medicine, University of Southern California, Los Angeles, CA, 90027

^*^To whom correspondence should be addressed.

This supplementary document is organized as follows. Section S1 lists the data sources used for construction of the whole-genome gene network that is used in TARGETgene. Section S2 details the network-based metrics used to identify potential therapeutic targets and driver cancer genes. Sections S3 presents some detail results of the first applications: identification of potential therapeutic targets from differentially expressed genes in several cancers. Sections S4 lists all references.

**S1 CONSTRUCTION OF THE GENE NETWORK**

Heterogeneous genomic and proteomic data (Table S1) were integrated using the RVM-based ensemble model reported in [**Wu et al., 2010**] in order to construct a whole-genome gene network. The nodes in this network represent all the genes of the human genome, and the probability between any two of them indicates the strength of their functional relationship, which can reveal the tendency of genes to operate in the same or similar pathways. The constructed gene network contains critical information about gene-gene functional relationships in biological pathways that can be used to explore diverse biological questions in health and disease, including exploring gene functions, understanding complex cellular mechanisms, and identifying potential therapeutic targets. TARGETgene uses this gene network to map and analyze potential therapeutic target at the systems level.

**S 1.1 Data Types Used for Construction of the Whole-Genome Gene Network**

Seventeen kinds of datasets (summarized in Table S1) were integrated to construct the gene network in this work. These data sources are from the following eight categories.

***Literature***

Automatic text mining techniques are generally used to extract co-occurrence gene relations from biological literature [**Li et al., 2006**]. In this work, however, we used expert-curated information from the NCBI, composed of genes and their corresponding cited literatures (<ftp://ftp.ncbi.nih.gov/gene/>). The numbers of co-citations for each gene pair was used to define the strength of the functional relationship for a gene pair.

***Gene Ontology***

Gene Ontology characterizes biological annotations of gene products using terms from hierarchical ontologies [**Ashburner et al., 2000**]. Three kinds of ontologies were used representing, the molecular function of gene products, their role in multi-step biological processes, and their localization to cellular components. We determined the functional relation of a gene pair by the following steps [**Rhodes et al., 2005; Qiu and Noble, 2008**]:

1. Identify all GO terms shared by the two genes.
2. Count how many other genes were assigned to each of the terms shared by the two genes.
3. Identify the shared GO terms with the smallest count. (In general, the smaller the count, the greater functional relationship between two genes.)
4. A functional value of a gene pair is computated as the negative logarithm of the smallest count.

**Table S1:** Data Features

| **Data Type** | **# of Genes** | **Data Source** |
| --- | --- | --- |
| Literature | 26,475 | Entrez Gene |
| Functional annotation | 14,667 | Ashburner *et al*., 2000. |
|  | 16,015 |  |
|  | 16,507 |  |
| Protein domain | 15,565 | Ng *et al*., 2003. |
| Protein-protein interaction and genetic interaction | 8,787 | Entrez Gene |
|  | 2,166 | Vastrik *et al*., 2007. |
|  | 6,982 | Gary *et al*., 2003. |
|  | 9,295 | Keshava Prasad *et al*., 2009. |
|  | 6,279 | Cline *et al*., 2007 |
|  | 1,959 | **Ewing *et al.*, 2007.** |
| Gene context | 9,159 | Kanehisa *et al*., 2010 |
|  | 11,303 | **Bowers *et al*., 2004** |
| Protein phosphorylation | 5,490 | Linding *et al*., 2008 |
|  | 3,205 | Yang *et al.*, 2008 |
| Gene expression profile | 19,777 | Obayashi *et al*., 2008 |
| Transcription regulation | 937 | Ferretti *et al*., 2007 |

***Protein-Protein Interactions and Genetic Interactions***

Experimental human protein-protein interactions were collected from diverse databases, including, NCBI, Reactome [**Vastrik et al., 2007**], BIND [**Gary et al., 2003**], HPRD [**Keshava Prasad et al., 2009**], and Cytoscape [**Cline et al., 2007**] (all were downloaded on December 2008). All the interactions are supported by different experiments, with most interactions in these sets derived from small-scale studies. Additional physical interactions were generated from published genome-scale screens using mass spectrometry analyses of affinity-purified protein complexes or high throughput yeast two hybrid (Y2H) assays. Since the experiments identifying the interactions can sometimes produce false-positives, we considered that number of different experiments of each gene pair as its confidence score. In addition, we also include protein-protein interactions from mass spectrometry data [**Ewing et al., 2007**].

***Protein Domain-Domain Interaction***

Proteins are known to interact with each other through protein domains, which represent modular protein subunits that are often repeated in various combinations throughout the genome. Thus, if two domains can physically interact, proteins containing these two domains are also likely to interact. In this work, we downloaded the predicted domain-domain interactions from the database InterDom (<http://interdom.i2r.a-star.edu.sg/>) [**Ng et al., 2003**]. These interactions were predicted based on protein structural information, and each interaction pair was assigned a confidence score. We assigned the score of each protein domain pair (inferred by InterDom) to all protein pairs containing them.

***Gene Context***

Comparative genome analyses of sequence information (Gene Context) have been successfully used to assign protein functions. The Prolinks database (<http://mysql5.mbi.ucla.edu/cgi-bin/functionator/pronav>) is a collection of these inference methods used to predict functional linkages between proteins [**Bowers et al., 2004**]. These include Gene Cluster, which uses genome proximity to predict functional linkage, Gene Neighbor, which uses both gene proximity and phylogenetic distribution to infer linkage, Rosetta Stone, which uses a gene fusion event in other organisms to infer functional relatedness, and Phylogenetic Profile which uses the presence or absence of proteins across multiple genomes to detect functional linkages [**Bowers et al., 2004**]. Internal Prolinks IDs of all genes were transferred to Entrez Gene IDs. The scores of gene pairs inferred by Prolinks were assigned as the Gene Context feature.

In addition, we also generated Phylogenetic profiles from the ortholog clusters in the KEGG database [**Kanehisa et al., 2010**], which describes the sets of orthologous proteins in 1111 organisms. In our work, we focused only on the 188 organisms with fully sequenced genomes [**Genome News Network, 2009**]. The phylogenetic profile of each gene consists of a string of bits which is coded as 1 and 0 to respectively indicate the presence and absence of its orthologous protein across the 188 organisms. The functional relationship of phylogenetic profiles for any two genes was then assessed using the mutual information (MI) values [**Date and Marcotte, 2003**]. A gene pair whose MI value is higher was considered as more confident functional interaction.

***Protein Phosphorylation***

Regulation of proteins by phosphorylation is one of the most common ways of regulation of protein function in a pathway. Protein kinases control cellular responses by phosphorylating specific substrates in a cascade of signaling processes. The NetworKIN database ([http://networkin.info](http://networkin.info/)) integrates consensus substrate motifs with context modeling to predict cellular kinase-substrate relationships based on the latest human phosphoproteome from the Phospho.ELM and PhosphoSite databases [**Linding et al., 2007; Linding et al., 2008**]. The database currently contains a predicted phosphorylation network of interactions involving 5,515 phospho-proteins and 123 human kinases. Ensemble IDs of all proteins were transferred to Entrez Gene IDs. The scores of gene pairs inferred by NetworKIN were directly assigned as the Protein Phophorylation feature. In addition, another data source of Protein Phophorylation, PhosphoPOINT [**Yang et al., 2008**], also provides 4,195 phospho-proteins, 518 serine/threonine/tyrosine kinases, and their corresponding protein interactions.

***Gene Expression***

Two genes in the same pathway are likely to have correlated gene expression profiles [**Tavazoie et al. 1999**]. Co-expression data were directly downloaded from COXPRESdb (<http://coxpresdb.hgc.jp/>), which was derived from publicly available GeneChip data [**Obayashi et al., 2008**]. It contains correlation data for 19,777 gene expression profiles in human.

***Transcription Regulation (Co-Regulation)***

Some genes in the same pathways are likely to be regulated by the same transcription regulators that bind to their regulatory elements. Gene co-regulation can be detected by ChIP-chip assays and may also be predicted by some computational approaches based on sequence motif information or phylogenetical conservation. In this work, the co-regulation data were downloaded directly from the PReMod database (<http://genomequebec.mcgill.ca/PReMod>), which describes more than 100,000 computationally predicted transcriptional regulatory modules within the human genome [**Ferretti et al., 2007**]. These modules represent the regulatory potential for 229 transcription factors families.

**S.1.2 Construction of the Gene Network using the RVM-based Ensemble Method**

These 17 diverse data sources were all used with the previously developed Relevance Vector Machines (RVM)-based ensemble approach [**Wu et al., 2010**] to compute the genetic functional associations (i.e., tendency of genes to operate in the same pathways) between all gene pairs given the input data features. The RVM-based model combined two ensemble approaches, AdaBoost [**Schapire and Singer, 1999**] and Sub-Feature [**Saar-Tsechansky and Provost, 2007**], to simultaneously address the two major problems associated with constructing a gene network: large-scale learning and massive missing data values. The Gold standard datasets for model building were generated from KEGG pathways. A complete explanation of RVM-based ensemble approach is provided in [**Wu et al., 2010**].

***The Data Matrix of the Gold Standard Set for Construction of A Gene Network***

Assume that a gene network is developed based on a set of N training examples (the Gold Standard Set),$\left\{x_{n},t_{n}\right\}_{n=1}^{N}$, where$x_{n}\in R^{d}$ (d is the number of features) represents a vector of measurements describing the nth training example, and $t_{n}\in\left\{0,1\right\}$ is a label vector indicating the classes to which the nth example belongs (1 and 0 denote interacting and non-interacting pairs respectively). The measurements of the N training examples,$\left\{x_{n}\right\}_{n=1}^{N}$, can be represented as a matrix as shown in the Figure S1 below. Each row presents a feature score vector *x_n_* of a gene pair that is composed of 17 feature scores of these two genes. For example, the feature score x_1,1_ is the # of co-citations of gene pair 1. Given an input *x_i_*, a gene pair *i* is then assigned as interacting (i.e., *t_i_^*^*=1) if the output *y_i_*(*x_i_*) ≥0 and as non-interacting (i.e., *t_i_^*^*=0) if the output *y_i_*(*x_i_*) <0.

As shown in the Table S1, different data features contain significantly varying degrees of coverage. These biological datasets present different types of pathway information. Thus there may be little overlap on gene pairs resulting in massive missing values (i.e. values of many x_i,j_ in the Figure S1 are missing.) on the order of tens of thousands or even more depending on the particular data sets.

*Figure S1: The score matrix of N training examples*

| x_1_ | x_1,1_ | x_1,2_ | … | x_1,14_ |
| --- | --- | --- | --- | --- |
| x_2_ | x_2,1_ | x_2,2_ | … | x_2,14_ |
| … | … | … | … | … |
| x_n_ | x_n,1_ | x_n,2_ | … | x_n,14_ |
| … | … | … | … | … |
| x_N_ | x_N,1_ | x_N,2_ | … | x_N,14_ |
|  | # of co-citation | correlation of gene expression | … | score of GO process |

**S2 NETWORK-BASED APPROACHES TO IDENTIFY IMPORTANT CANCER-RELATED GENES**

Based on this constructed gene network, TARGETgene identifies potential therapeutic targets using one of two network-based metrics: 1) hub score, which uses a centrality measure to identify hub genes in a tumor-specific network, or 2) seed gene association score, which quantifies each genes association with known cancer (disease) genes.

**S 2.1 Identification using Network Centrality Metrics**

In view of the complexity in cancers, potential therapeutic targets can be those genes/proteins that have a critical role in regulating multiple pathways or maintaining those malignant phenotypes. Recently, cancer-associated genes are found more likely to be signaling proteins that act as signaling hubs, actively sending or receiving signals through multiple signaling pathways [**Cui et al., 2007**]. In addition, under the modular structure of biological networks, intermodular hubs are found to be more associated with cancer phenotypes than intramodular hubs, since intermodular hubs interact with other intramodular hubs temporally and spatially that in turn fulfill different specific molecular functions [**Taylor et al., 2009**]. Therefore, potential therapeutic targets can be those hub genes in a tumor-specific network. A tumor-specific network can be generated by directly mapping the candidate gene (e.g., differentially expressed genes in a tumor) to the constructed gene network. Two centrality measurements provided in TARGETgene can qunatify the tendency of a gene to be a hub in the tumor-specific network. All candidate genes in the tumor-specific network are ranked based on their centrality measurement in the tumor-specific network. Those highly ranked hub genes can be considered as potential therapeutic targets.

Topological measures of centrality, such as total degree [**Freeman, 1977**], betweenness [**Freeman, 1977**], closeness [**Freeman, 1979**], and eigenvector centrality [**Newman, 2003**] are typically used to determine hub genes (central nodes) in a binary network (i.e, unweighted network). However, since most gene pairs in a tumor-specific networ have weighted linkages, betweenness and closeness, which are limited to calculation of the shortest path between any two gene pairs, are not used for calculating centrality in TARGETgene. Instead, the centrality metrics, weighted degree centrality and weighted eigenvector centrality [**Barrat et al., 2004; Newman, 2004**] are used in TARGETgene and briefly discussed below.

***Weighted degree centrality***

In a weighted network, it is intuitive to consider a definition of total degree that is based on the strength of nodes in terms of the total weight of their connections [**Barrat et al., 2004; Newman, 2004**].

$d_{i}=\sum_{j=1}^{n} w_{i,j}$ (S1)

where *d_i_* is the centrality measurement of gene *i*, *w_i,j_* is the functional relationship between gene *i* and gene *j* in the network, and *n* is the number of differently expressed genes. Highly weighted nodes (larger *d_i_*) are more central.

***Weighted Eigenvector centrality***

Weighted degree centrality only counts local impact of a gene through its direct connections in the network. Thus, some bottleneck hubs [**Yu et al., 2007**] that have few connections with other nodes but acts key connectors in a network thus are not able to be determined using weighted degree centrality. Thus, eigenvector centrality that can count global importance of a gene in the network through both its direct and indirect connections with other genes is also provided in TARGETgene. Eigenvector centrality is closely related to “PageRank”, a similar centrality measure used in web search engines. The eigenvector centrality *e_i_* of a vertex in a weighted network is proportional to the weighted sum of the centralities of the vertex’s neighbors. Thus a vertex can acquire high centrality either because it is connected to a many others or because it is connected to others that themselves highly central [**Newman, 2004**]. We can write

$e_{i}=\lambda^{-1}\sum_{j=1}^{n} w_{i,j}e_{j}$ (S2)

where $\lambda$ is a constant. Using matrix notation, Eq. (S2) can be written $\lambda E=WE$, so that E is an eigenvector of the adjacency weighted matrix W of a weighted network. The eigenvector centrality of all vertexes is the eigenvector corresponding to the max eigenvalue.

**S 2.2 Association with Seed Genes (Known Cancer Genes)**

Genes associated with similar disease phenotypes tend to be interconnect in a biological network (i.e., participate in the same molecular pathway or the same protein complexes). Based on this concept, several network-based computational approaches [**Franke et al., 2006; Köhler et al., 2008; Chen et al., 2009; Linghu et al., 2009**] have been proposed to predict novel disease genes. Given a set of known genes of a disease (i.e. seed genes), functional associations (linkages) of other genes with these seed genes in biological networks can be calculated. Genes that are found to be more associated with the known disease genes are more likely involved in the disease process.

Therefore, TARGETgene also allows users to identify important cancer genes or potential therapeutic targets by associating them with user-defined seed genes (e.g., known cancer genes) in the gene network. More specifically, the importance of each candidate gene is calculated as summation of its direct functional association with those seed genes.

$c_{i}=\sum_{j=1}^{m} w_{i,j}$ (S3)

where *c_i_* is the degree of association of gene *i* with seed genes, *m* is the number of seed genes, and *w_i,,j_* is the functional association of gene *i* to seed gene *j* in the constructed gene network. Genes with more associations with all the seed genes (i.e., larger c values) are likely to play more important roles in the cancer and can be potential therapeutic targets.

**S3 EXAMPLE 1: IDENTIFICATION OF POTENTIAL THERAPEUTIC TARGETS FROM DIFFERENTIALLY EXPRESSED GENES**

**S3.1 Rank Genes Based On Their Weighted Degree Centrality in the Tumor-Specific Network**

In this example, TARGETgene was applied in turn to each of three cancer types: Her2-positive breast cancer, colon cancer, and Lung Adenocarcinoma. Human Exon datasets in the Affymetrix platform for the three cancer types were collected from the National Center for Biotechnology Information Gene Expression Omnibus (GEO) [**Barrett et al., 2007**]. There are 10 and 20 tumor/normal paired specimens in Colon Cancer [**Affymetrix sample data of exon array**] and Lung Adenocarcinoma (GSE12236) [**Xi et al., 2008**], respectively. In addition, the case study of Breast Cancer includes 35 samples from patients with HER2 positive and three samples from normal breast tissues (GSE16534) [**Lin et al., 2009**]. Subsequent data analyses were done using Partek Genomic Suite 6.3 (Partek Inc.). The RMA (Robust Multichip Analysis) algorithm [**Irizarry *et. al.*, 2003**] was used to do background correction, normalization and summarization. Exon-level data in each cancer type was then filtered to include only those probesets that represent 17,800 RefSeq genes and full-length GenBank mRNAs. Any effect of different microarray processing was removed using a batch removal tool of Partek Genomic Suite. ANOVA p-values and fold changes of gene expression in cancer samples against normal tissues were calculated. Finally, using a criteria of P<0.01 in the ANOVA analysis, 5203, 5,153 and 6,203 differentially expressed genes were identified in case studies of colon, breast, and lung cancer, respectively.

Differentially expressed genes in each cancer type were all ranked based on the extent of their weighted degree of centrality (Section S2.1) in a tumor-specific network, which was generated by mapping the differentially expressed genes in each cancer type to the constructed gene network (Section S1). Figure S2.a, b, and c list the top 10 highest ranked genes for each of the three cancer types as shown in the Gene Panels of TARGETgene. The complete ranking list of genes for each of the three cancer types can be obtained by running TARGETgene using the candidate genes list stored in the examples files and selecting the weighted degree centrality ranking option. The results show that a number of important cancer genes for each cancer type are ranked highly by TARGETgene including: AKT1 (#1), SRC (#10), ERBB2 (#25), and ESR2 (#56) in breast cancer; MYC (#174), CTNNB1 (#119), APC (#116), and DCC (#195) in colon cancer; KIT (#30), ERBB2 (#31), PPARG (#77), and PTEN (#157) in lung cancer. In addition, TARGETgene also ranks several genes highly (in the top 10%) that were recently identified as cancer-related genes in each cancer type. For example, in breast cancer we ADAM12 ( rank #153) and MAP3K6 ( rank #205) were recently reported to be associated with breast cancer oncogenesis [**Sjoblom et al., 2006; Wood et al., 2007**].

Moreover, many genes that have never been identified in each type of cancer are also ranked highly. These genes could be subject *in vitro* and *in vivo* study to evaluate their importance in each cancer type. Several of these have been identified by RNAi screens (Section S3.2.4 presents details on evaluation of predictions based on RNAi screens). For example, in colon cancer, RIPK2 and ENC1 (ectodermal-neural cortex) have a TARGETgene rank of 8 and 257, respectively. RIPK2 encodes a member of the receptor-interacting protein (RIP) family of serine/threonine protein kinases. It is also a potent activator of NF-kappaB and inducer of apoptosis in response to various stimuli [**Tao et al., 2009**]. ENC1 activates p53 tumor suppressor protein and induces cell cycle arrest or apoptosis [**Polyak et al., 1997**]. It also has been shown to be involved in oncogenesis of brain [**Seng et al., 2009**] and breast cancer [**Seng et al., 2007**]. In breast cancer, PIK3R2 (phosphoinositide-3-kinase, regulatory subunit 2 beta) and CIT (citron) have a TARGETgene rank of 37 and 115, respectively. PIK3R2, with a 3.31 fold change in gene expression of breast cancer tissues, has been shown to be functionally involved in several cancer related pathways, such as the PI3K/Akt pathway [**Radhakrishnan et al., 2008**], and also associated with several other cancer types, such as ovarian cancer [**Zhang et al., 2007**]. CIT (citron), with a 3.06 fold change in gene expression in breast cancer tissues is a kinase that has been identified to be associated with the cell cycle [**Liu et al., 2003**]. In lung adenocarcinoma, MAPK13 and CBLC (Cas-Br-M (murine) ecotropic retroviral transforming sequence c) have TARGETgene ranks 19 and 173, respectively. MAPK13 is involved in a wide variety of cellular processes such as proliferation, differentiation, transcription regulation and development. MAPK13 has also been found to be a downstream carrier of the PKCdelta-dependent death signaling [**Efimova et al., 2004**]. CBLC has been reported to interact with AIP4 to cooperatively down-regulate EGFR signaling [**Courbard et al., 2002**]. In addition, CBLC also been shown to be a negative regulator of receptor tyrosine kinase Met signaling in B cells and to mediate ubiquitination and thus proteosomal degradation of Met, with a role in Met-mediated tumorigenesis [**Taher et al., 2002**]

Figure S2. Screen shots from Gene Panel for each cancer type

**S3.2 Evaluation of Predictions**

TARGETgene also compares its resulting ranked genes to several benchmark gene sets, including the set of curated cancer genes, the set of genes cited in cancer literature, and the set of target genes detected by RNAi screens. Receiver Operating Characteristic (ROC) Curves are used for this evaluation.

***S3.2.1 Evaluation of Predictions using Known Cancer Genes***

The 1,186 curated cancer genes downloaded from the CancerGenes database [**Higgins et al., 2006**] are first used to evaluate if they are highly ranked by TARGETgene. These cancer genes, however, are not classified to any specific cancer type. For each cancer type, we therefore treat those genes as specific to a cancer type if they are cited by literature source related to that cancer type (Pubmed data on Dec. 2008). The curated cancer genes are considered as positive instances while other remaining genes are treated as negative instances. Figure S3.a shows TARGETgene’s prediction performance for each cancer type, evaluated using ROC curves and AUC. The high AUC values of TARGETgene’s prediction in each cancer type (all AUC > 0.85) indicate that most of known cancer genes tend to be ranked highly. (This result also reveals that the human gene network constructed by the RVM-based model contains critical pathway information and can successfully be used to identify other important cancer genes.)

Genes that are cited by the literature of each cancer type are also used for evaluation. In this work, all Pubmed IDs of literature related to colon cancer, breast cancer, and lung adenocarcinoma were first downloaded from Pubmed on Dec. 2008. For each gene, we calculated the number of citations related to each cancer type by mapping the extracted Pubmed IDs to the gene citation information from Entrez Gene (ftp://ftp.ncbi.nih.gov/gene/), composed of genes and their corresponding cited literature. The evaluation was also based on ROC curves. Figure S3.b shows the ROC curves for the three cancer types in which genes are selected as the benchmark genes if they are cited by at least one cancer literature. The AUC values of the ROC curves for TARGETgene’s predictions are great than 0.7 for each cancer type. It is expected that the resulting AUC’s are uniformly lower when compared to those obtained using the curated cancer genes as the benchmark, because literature citation data are noisy. The results using literature citation also depend on the number of citations (set at 1 in the results shown in Figure S3.b). In addition, as the citation cutoff number used increases (Figure S4.a-c) so do the resulting TARGETgene AUC values, indicating that genes with more citations (presumably because they are more extensively studied) also have a higher TARGETgene ranking (Figure S5.a-c). Spearman's rank correlation is also used to assess correlation between citation number and TARGETgene ranking. The resulting correlations for colon, breast and lung cancer are 0.2665, 0.3658, and 0.2927, respectively, which are all significantly higher than random expectation (P~=0.000). Recall that TARGETgene ranks many novel genes without any previous literature citations highly, which depresses the Spearman rank correlation coefficient. Nevertheless, this provides further evidence genes highly ranked by TARGETgene are also are cited more in the cancer literature.

**Figure S3.** ROC curve performance evaluation (true positive rate – TPR, versus false positive rate – FPR) of TARGETgene using curated cancer genes (a) and genes cited by cancer literature (one or more citations) (b).

**Figure S4.** ROC curve performance evaluation (true positive rate – TPR, versus false positive rate – FPR) of TARGETgene using genes cited by cancer literature with different citation number cutoff values of 1, 5 and 10.

**Figure S5.** Number of cancer literature citation of genes vs TARGETgene gene ranks (Gene Ranking Block) in the predictions of each cancer type.

***S3.2.2 Evaluation of Predictions using Gene Function Annotations***

Gorilla [**Eden et al., 2009**], a gene ontology enrichment analysis tool, was applied to identify enriched GO terms that appear densely at the top of TARGETgene’s ranked gene lists for each of the three cancer types. Many of identified GO process terms are known cancer-related biological processes. The examples of indentified biological process terms include, regulation of cell death (GO:0010941), regulation of apoptosis (GO:0042981), regulation of cell proliferation (GO:0042127), regulation of cell migration (GO:0030334), angiogenesis (GO:0001525; GO:0060055), and regulation of cell differentiation (GO:0045595). Interestingly, several biological processes related to new hallmarks of cancers [**Luo et al., 2009**] are also identified. They are DNA damage (GO:0006974, GO:0042770), oxidative stress (GO:0070482), evading immune surveillance (GO:0002682; GO:0002684), metabolic stress (GO:0006796; GO:0006793), mitotic stress (GO:0007059; GO:0007346), and proteotoxic stress (GO:0009408; GO:0051603). These results indicate that genes highly ranked by TARGETgene are involved in multiple cancer-related biological processes and pathways.

Several types of molecules, such as signaling kinases, receptor tyrosine kinases, and transcription factors are often proposed as possible molecular targets in cancers [**Shawver et al, 2002; Sawyers, 2004; Krause et al., 2005; Frank, 2009**]. For example, protein kinases are enzymes that modify other proteins by chemically adding phosphate groups to them (protein phosphorylation). Protein phosphorylation has proven to be an important driving force in cellular signaling. Protein kinases can impact many cellular processes through their ability to control protein-protein interactions, complex formation, enzyme activity and protein degradation and translocation [**Seet et al., 2006**]. We find that many kinase, receptor, and transcription factor related GO function terms are enriched in highly-ranked genes in TARGETgene (Figure S6). The examples of indentified molecular function terms include protein serine/threonine kinase activity (GO:0004674), protein tyrosine kinase activity (GO:0004713), kinase binding (GO:0019900), growth factor receptor binding (GO:0070851), transcription regulator activity (GO:0030528), and transcription factor binding (GO:0008134). The results indicated that many of the genes highly ranked by TARGETgene are kinase, receptor, and transcription factor related genes.


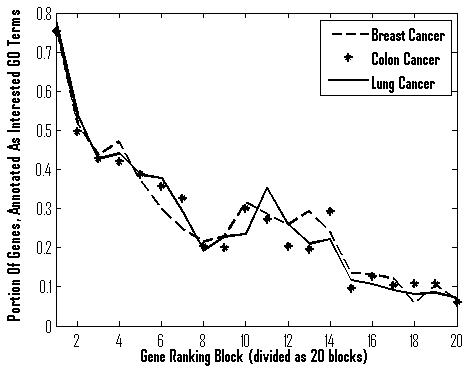


Figure S6. Proportion of genes related to kinase, receptor, and transcription factors vs TARGETgene gene ranks (Gene Ranking Block) in the predictions of each cancer type.

***S3.2.3 Evaluation using The Results of RNAi Screens***

High-throughput RNA interference (RANi) screens are a powerful tool for genome-wide knockdown of specific gene products or perturbation of gene expression. The phenotypic results from the screen can be monitored by assaying for specific alterations in molecular and cellular endpoints, such as promoter activation, cell proliferation and viability [**Iorns et al., 2007**]. RNAi screens have recently been shown to be a promising tool to discover new targets for the treatment of several cancers. Therefore, data from RNAi screens can be applied to evaluate the performance of the predictions from TARGETgene. Effective targets of each cancer type detected by RNAi screens were all downloaded from GenomeRNAi, a database for cell-based RNAi phenotypes [**Gilsdorf et al., 2009**]. This database contains phenotypes from a number of cell-based RNA interference screens in human cells. We selected the RNAi screens for the three cancer types whose phenotype is related to cell viability. The data sources of RNAi screens used in this work are summarized in **Table S2a-c**. Since lung adenocarcinoma is a type of non-small cell lung cancer (NSCLC), the result of RNAi screens in NSCLC cell lines were used for evaluation. The evaluation of prediction performance was assessed using ROC curve. In each case of cancer type, the effective targets detected by RNAi screens are treated as positive instances while others genes are treated as negative instance.

**Table S2a: Data Sources of RNAi Screens in Breast Cancer Cell Lines**

| **Reference** | **Assay** | **Cell Lines** | **Number of**  **Detected Targets** |
| --- | --- | --- | --- |
| Simpson et al., 2008 | Cell migration and viability | MCF-10A | 66 |
| Schlabach et al., 2008 | Cell viability | HCC1954 | 176 |
| Silva et al., 2008 | Cell viability | MCF-10A; MDA-MB-435 | 172 |
| Swanton et al., 2007 | Cell viability | MDA-MB-231 | 45 |
| Turner et al., 2008 | Cell viability | CAL51 | 24 |
| Iorns et al., 2009 | Cell viability | MCF-7 | 20 |
| Brummelkamp et al., 2006 | Cell viability | MCF-7 | 13 |
| Total Number of detected targets | | | 441 |

**Table S2b**: Data Sources of RNAi Screens in Colon Cancer Cell Lines

| **Reference** | **Assay** | **Cell Lines** | **Number of**  **detected targets** |
| --- | --- | --- | --- |
| Schlabach et al., 2008 | Cell viability | DLD1; HCT116 | 243 |
| Moffat et al., 2006 | Mitotic index/Cell viability | HT29 | 128 |
| Swanton et al., 2007 | Cell viability | HCT-116 | 45 |
| Firestein et al., 2008 | Wnt signaling/Cell viability | DLD1; HCT116 | 9 |
| Total Number of detected targets | | | 271 |

**Table S2c**: Data Sources of RNAi Screens in NSCLC Cell Lines

| **Reference** | **Assay** | **Cell Lines** | **Number of**  **Detected Targets** |
| --- | --- | --- | --- |
| Swanton et al., 2007 | Cell viability | A549 | 45 |
| Ji et al., 2007 | Cell viability | A549 | 10 |
| Total Number of detected targets | | | 55 |

The result is shown in Figure S7. The high AUC in each cancer type indicates that the most effected targets identified in the genome-wide RNAi screens tend to be ranked highly by TARGETgene. Some highly ranked genes have been shown to play an important role in oncogenesis in each of the three cancer type, such as AKT1 (#1) in Breast Cancer, MET (#107) in Colon Cancer, and PTEN (#157) in NSCLC. Most interestingly, we also found that many novel targets (i.e., no citation related to the specific cancer type based on PubMed in Dec. 2008) detected by RNAi screens are also ranked highly by TARGETgene. For example, CASK (calcium/calmodulin-dependent serine protein kinase) and RUVBL1 (RuvB-like 1) are ranked 161 and 433, respectively in the prediction of breast cancer. Such results provide support from cell line models for the ability of TARGETgene to identify novel therapeutic targets in cancers. This also suggests the possibility of combination of RNAi and network-based screens for therapeutic target identification as discussed.


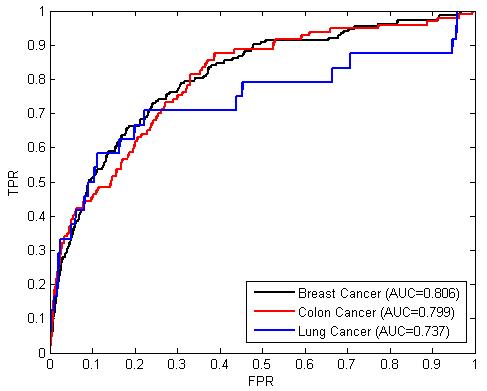


**Figure S7** TARGETgene prediction performances (true positive rate – TPR, versus false positive rate – FPR) evaluated by the results of RNAi screens (cell viability).

***S3.3 Mapping the Predictions to Drug-Target Information***

Recently, information on drugs/compunds and their targets that have been approved or are under evaluation for use in cancer treatment, have become available electronically and accessible through several public databases. This information is used by TARGETgene to report those drugs/compounds that could have action of the targets identified by TARGETgene. In this work, the information of drugs/compounds and their targets were compiled from DrugBank [**Knox et al., 2011**], PharmGKB [**Hodge et al., 2007**], and Therapeutic Target Database [**Zhu et al., 2009**] on Dec. 2010. The database extracted from these sources and used by TARGETgene contains nearly 4800 drug entries, including 1,350 FDA-approved small molecule drugs, 123 FDA-approved biologics (protein/peptide) drugs, 71 nutraceuticals, and 3,243 experimental drugs, as well as approximately 6,000 drug-target relationships.

After mapping the information of drugs/compounds and their targets to the ranked gene lists from TARGETgene, we found that many genes highly rank by TARGETgene are targets for some drugs that have already been in clinical trials or have been used for treatment of the three cancer types. Other identified drugs and compounds that are not used in clinical trials have also shown anti-cancer effect and could thus be considered as potential novel drug for these cancers. Table S3.a-c lists some of these drugs and compounds whose targeted genes are overexpressed and highly ranked by TARGETgene in each cancer type. In the case of breast cancer, Trastuzumab and Lapatinib have been approved for HER2 positive Breast cancer, and their main target erbB2 is very highly ranked by TARGETgene (and up-regulated). Other endocrine treatments for ER-postive breast cancer, such as Taxmoxifen, are not included because its main target ESR1 and ESR2 are not overexpressed in our analysis. Several other drugs, such as Dasatinib, UCN-01, Celecoxib, Flavopiridol, and Vorinostat, have already been in clinical trials for the treatment of breast cancer. Some of their targets are highly ranked by TARGETgene. Moreover, other drug/compounds have been shown to have anti-tumor effects and could be considered as potential novel drugs for the treatment in breast cancer, such as Alsterpaullone and Olomoucine. In addition, two naturally occurring compounds, melatonin and vitamin D (Calcidiol), are also identified by TARGETgene. Melatonin, a naturally occurring compound found in organisms, can regulate the circadian rhythms of several biological functions. Recently, a clinical trial involving a total of 643 cancer patients using melatonin found a reduced incidence of death [**Mills et al., 2005**]. A study showed that women with low melatonin levels have an increased risk for breast cancer [**Navara and Nelson, 2007**]. Vitamin D receptors have been found in up to 80% of breast cancers, and vitamin D receptor polymorphisms have been associated with differences in survival [**Buras et al., 1994; Friedrich et al., 2002; Diesing et al., 2006**]. Active vitamin D compounds (Calcidiol; Calcitriol) also have been identified for their antiproliferative effects in breast cancer cells [**Costa et al., 2009; Köstner et al., 2009**], although the detail mechanisms are still unclear. In summary, these results provide some further evidence that genes that are highly ranked by TARGETgene be potential therapeutic targets.

**Table S3.a Selected Drugs Targeting High-Ranked Genes in Breast Cancer Identified by TARGETgene**

| **Drugs/Compounds** | **Gene and Ranking** | **Fold Changes in Cancer** | **Literatures Of**  **Breast Cancer Treatment** |
| --- | --- | --- | --- |
| Dasatinib (E)* | SRC(#10) | 2.623 | Fornier et al., 2011  Herold et al., 2011 |
| Celecoxib (A)* | PDPK1 (#14) | 2.917 | Fujii et al., 2008 |
| Staurosporine (UCN-01) (E)* | PDPK1 (#14)  MAPKAPK2 (#62)  CSK (#19)  GSK3B (#84) | 2.917  2.138  3.724  2.130 | Koh et al., 2002  Hawkins et al., 2005 |
| Flavopiridol (E)* | CDK5 (#41)  CDC2 (#108)  CDK4 (#50) | 4.640  4.382  2.092 | Fornier et al., 2007  Witters et al., 2004 |
| Alsterpaullone (E) | CDK5 (#41)  GSK3B (#84)  CDC2 (#108) | 4.640  2.130  4.382 | Kohfeld et al., 2007 |
| Olomoucine (E) | CDC2 (#108)  CDK5 (#41) | 4.382  4.640 | Wesierska-Gadek et al., 2004 |
| Trastuzumab (A)*** | ERBB2 (#25) | 46.856 | Wardley et al., 2009.  Kaufman et al., 2009 |
| Lapatinib (A)*** | ERBB2 (#25) | 46.856 | Frampton, 2009.  Esteva et al., 2009 |
| Dexrazoxane (A)*** | TOP2A(#302) | 10.965 | Gligorov and Lotz, 2008 |
| Lithium (A) | GSK3B (#84) | 2.130 | Farina et al., 2009. |
| Melatonin (A) | CALR(#651) | 1.778 | Navara and Nelson, 2007 |
| Calcidiol (A) | VDR (#241) | 3.358 | Costa et al., 2009  Köstner et al., 2009 |
| Vorinostat (A)* | HDAC3 (#307)  HDAC1 (#497)  HDAC2 (#564) | 2.336  2.286  2.520 | Luu et al., 2008 |
| Geldanamycin (17-AAG) (E)* | HSP90B1 (#258)  HSP90AA1 (#275) | 1.779  1.920 | Beliakoff and Whitesell, 2004  Perotti et al., 2008 |
| Arsenic trioxide (A)* | AKT1 (#1)  CCND1 (#418) | 4.566  3.663 | Li et al., 2004  Ye et al., 2005 |

**Note**: 1.Approved drugs are denoted as ‘A’

2.Experimental compounds are denoted as ‘E’

3.Drugs have been approved for the treatment of Breast Cancer are marked with ***

4.Drugs in clinical trials for Breast Cancer are marked with *

**Table S3.b Selected Drugs Targeting High-Ranked Genes in Colon Cancer Identified by TARGETgene**

| **Drugs/Compounds** | **Gene & Ranking** | **Fold Changes in Cancer** | **Literatures Of**  **Colon Cancer Treatment** |
| --- | --- | --- | --- |
| Celecoxib (A)* | PDPK1 (#7) | 1.256 | Yang et al., 2010 |
| Sorafenib (A)* | KDR (#25)  PDGFRB (#40) | 1.203  1.614 | Walker et al., 2009 |
| Sunitinib (A)* | KDR (#25)  PDGFRB (#40) | 1.203  1.614 | Blesa and Pulido, 2010 |
| Dasatinib (A)* | PDGFRB (#40)  EPHA2 (#61) | 1.614  1.440 | Kopetz et al., 2009 |
| Etoposide (A)* | TOP2A (#291) | 1.748 | Chamberlain et al., 2006  Schonn et al., 2009 |
| Vorinostat (A)* | HDAC3 (#293)  HDAC2 (#634) | 1.234  1.427 | Walker et al., 2009  Fakih et al., 2009 |
| Bevacizumab (A)*** | FCGR2A (#171)  FCGR3A (#190)  VEGFA (#872) | 1.503  1.832  1.709 | Giantonio et al., 2007  Hurwitz et al., 2004 |
| Cetuximab (A)*** | FCGR2A (#171)  FCGR3A (#190) | 1.503  1.832 | Zhang et al., 2007 |
| Atorvastatin (A)* | AHR(#540) | 1.375 | Yang et al., 2010  Poynter et al., 2005 |
| Imatinib (A)* | PDGFRB (#40)  DDR1 (#145) | 1.614  1.308 | Kitadai et al., 2006  Mueller et al., 2007 |
| SU9516 (E) | CDK5 (#23)  CDC2 (#102)  CDK2 (#47) | 1.302  1.247  1.394 | Takagi et al., 2008  Lane et al., 2001 |
| Flavopiridol (E)* | CDK5 (#23)  CDK7 (#28)  CDC2 (#102)  CDK4 (#32)  CDK6 (#45)  CDK2 (#47) | 1.302  1.171  1.247  1.666  1.515  1.394 | Ambrosini et al., 2008.  Newcomb, 2004 |
| Staurosporine  (UCN-01) (E)* | PDPK1 (#7)  GSK3B (#70)  CDK2 (#47) | 1.256  1.208  1.394 | Hotte1 et al., 2006 |
| Epirubicin (A)* | TOP2A (#291) | 1.748 | Goff et al., 2008 |

**Table S3.c Selected Drugs Targeting on High-Ranked Genes in Lung Cancer Identified by TARGETgene**

| **Drugs/Compounds** | **Gene & Ranking** | **Fold Changes in Cancer** | **Literatures Of**  **Lung Cancer Treatment** |
| --- | --- | --- | --- |
| Bevacizumab (A)*** | FCGR2A (#252)  FCGR3A (#291)  C1QA (#747)  C1QB (#1567) | 1.965  2.072  1.890  1.825 | Johnson et al., 2004  Reck et al., 2009 |
| Sorafenib (A)* | KDR (#50)  KIT (#30)  FLT4 (#34)  PDGFRB (#69) | 2.100  2.440  1.526  1.937 | Blumenschein et al., 2009  Scagliotti et al., 2009 |
| Sunitinib (A)* | FLT1 (#12)  CSF1R (#16)  KDR (#50)  KIT (#30)  FLT4 (#34)  PDGFRB (#69)  PDGFRA (#149) | 1.925  1.560  2.100  2.440  1.526  1.937  1.492 | Pal et al., 2010  Socinski et al., 2008 |
| Cetuximab (E)* | FCGR2A (#252)  FCGR3A (#291)  C1QA (#747)  C1QB (#1567) | 1.965  2.072  1.890  1.825 | Hanna et al., 2006  Horn and Sandler, 2009 |
| Trastuzumab (A)* | FCGR2A (#252)  FCGR3A (#291)  C1QA (#747)  C1QB (#1567) | 1.965  2.072  1.890  1.825 | Ferrone and Motl, 2003  Cappuzzo et al., 2006 |
| Dasatinib (A)* | FYN (#5)  ABL1 (#1)  KIT (#30)  PDGFRB (#69)  STAT5B (#212) | 1.691  1.524  2.440  1.937  1.581 | Haura et al., 2010  Li et al., 2010 |
| Sulindac (A)* | MAPK3 (#55)  PTGS1 (#2914)  PTGS2 (#1213) | 1.618  1.841  2.903 | Attia et al., 2008  Jin et al., 2008 |
| Staurosporine  (UCN-01) (E) | PRKCQ (#2) | 1.409 | Edelman et al., 2007  Wang et al., 2009 |

**S4. REFERENCE**

Ambrosini, G. et al. (2008) The cyclin-dependent kinase inhibitor flavopiridol potentiates the effects of topoisomerase I poisons by suppressing Rad51 expression in a p53-dependent manner. Cancer Res. 68(7): 2312-20.

Ashburner M et al. (2000) Gene Ontology: tool for the unification of biology. *Nature Genetics* 25: 25–9.

Attia, S. et al. (2008) Phase I/II study of vinorelbine and exisulind as first-line treatment of advanced non-small cell lung cancer in patients at least 70 years old: a wisconsin oncology network study. J Thorac Oncol. 3(9): 1018-25.

Barrat, A. et al. (2004) The architecture of complex weighted networks. *Proc Natl Acad Sci U S A.* 101(11): 3747-52.

Barrett, T. et al., (2007) NCBI GEO: Mining tens of millions of expression profiles databases and tools update. Nucleic Acids Res. 35: D760.

Beliakoff, J. and Whitesell, L. (2004) Hsp90: an emerging target for breast cancer therapy. Anticancer Drugs 15(7): 651-62.

BIG 1-98 Collaborative Group et al. (2009) Letrozole therapy alone or in sequence with tamoxifen in women with breast cancer. N Engl J Med. 361(8): 766-76.

Blesa, J. M. and Pulido, E. G (2010) Colorectal cancer: response to sunitinib in a heavily pretreated colorectal cancer patient. *Anticancer Drugs* Suppl 1: S23-6.

Blumenschein, G. R. Jr. (2009) Phase II, multicenter, uncontrolled trial of single-agent sorafenib in patients with relapsed or refractory, advanced non-small-cell lung cancer. J Clin Oncol. 27(26): 4274-80.

Bowd, C. et al. (2005) Relevance Vector Machine and Support Vector Machine Classifier Analysis of Scanning Laser Polarimetry Retinal Nerve Fiber Layer Measurements, Investigative Ophthalmology and Visual Science 46: 1322-9.

**Bowers**, P.M. et al. (2004) Prolinks: a database of protein functional linkages derived from coevolution. Genome Biology **5:** R35.

Brummelkamp, T. R. et al. (2006) An shRNA barcode screen provides insight into cancer cell vulnerability to MDM2 inhibitors. *Nat Chem Biol*. 2(4): 202-6.

Buras, R. R. et al. (1994) Vitamin D receptors in breast cancer cells. Breast Cancer Res Treat 31: 191-202.

Cappuzzo, F. et al. (2006) HER2 mutation and response to trastuzumab therapy in non-small-cell lung cancer. N Engl J Med. 354(24): 2619-21.

Cerami, E. et al. (2010) Automated network analysis identifies core pathways in glioblastoma. PLoS One 5(2): e8918.

Chamberlain, M. C. et al. (2006) Phase II trial of intracerebrospinal fluid etoposide in the treatment of neoplastic meningitis. Cancer 106(9): 2021-7.

Chen, J. et al. (2009) Disease candidate gene identification and prioritization using protein interaction networks. BMC Bioinformatics 10:73.

Cline, M. S. et al. (2007) Integration of biological networks and gene expression data using Cytoscape. *Nat Protoc.* 2(10): 2366-82.

Costa, J. L. et al. (2009) Anti-proliferative action of vitamin D in MCF7 is still active after siRNA-VDR knock-down. BMC Genomics 10:499.

Courbard, J. R. et al. (2002) Interaction between two ubiquitin-protein isopeptide ligases of different classes, CBLC and AIP4/ITCH. J Biol Chem. 277(47): 45267-75.

Cui, Q. et al. (2007) A map of human cancer signaling. Mol Syst Biol. 3: 152.

Date, S. V. and Marcotte, E.M. (2003) Discovery of uncharacterized cellular systems by genome-wide analysis of functional linkages, *Nat. Biotechnol.* 21: 1055–1062.

Diesing, D.et al. (2006) Vitamin D--metabolism in the human breast cancer cell line MCF-7. Anticancer Res. 26(4A): 2755-9.

Ding,L. et al. (2008) Somatic mutations affect key pathways in lung adenocarcinoma, Nature 455 (7216):1069-75.

Edelman, M. J. et al. (2007) Phase I and pharmacokinetic study of 7- hydroxystaurosporine and carboplatin in advanced solid tumors. Clin Cancer Res. 13(9): 2667-74.

Eden, E. et al. (2009) GOrilla: A Tool for discovery and visualization of enriched GO terms in ranked gene Lists. BMC Bioinformatics 10: 48.

Efimova, T. et al. (2004) Protein kinase Cdelta regulates keratinocyte death and survival by regulating activity and subcellular localization of a p38delta-extracellular signal-regulated kinase 1/2 complex. Mol Cell Biol. 24(18): 8167-83.

Esteva, F. J. et al. (2009) Molecular predictors of response to trastuzumab and lapatinib in breast cancer. Nat Rev Clin Oncol. Dec 22.

Ewing, R. M. et al. (2007) Large-scale mapping of human protein-protein interactions by mass spectrometry. *Mol Syst Biol.* 3: 89.

Fakih, M. G. et al. (2009) A phase I, pharmacokinetic and pharmacodynamic study on vorinostat in combination with 5-fluorouracil, leucovorin, and oxaliplatin in patients with refractory colorectal cancer. Clin Cancer Res. 15(9): 3189-95.

Farina, A. K. et al. (2009) Post-transcriptional regulation of cadherin-11 expression by GSK-3 and beta-catenin in prostate and breast cancer cells. PLoS One 4(3): e4797.

Ferretti, V. et al. (2007) PReMod: a database of genome-wide mammalian cis-regulatory module predictions. *Nucleic Acids Res.* 35(Database issue): D122-6.

Ferrone, M. and Motl, S. E. (2003) Trastuzumab for the treatment of non-small-cell lung cancer. Ann Pharmacother. 37(12): 1904-8.

Firestein, R. et al. (2008) CDK8 is a colorectal cancer oncogene that regulates beta-catenin activity. *Nature* 455(7212): 547-51.

Fornier, M. N. et al. (2007) Phase I dose-finding study of weekly docetaxel followed by flavopiridol for patients with advanced solid tumors. Clin Cancer Res. 13(19): 5841-6.

Fornier, M.N. et al. (2011) A phase I study of dasatinib and weekly paclitaxel for metastatic breast cancer. Ann Oncol. [Epub ahead of print]

Frampton, J. E. (2009) Lapatinib: a review of its use in the treatment of HER2-overexpressing, trastuzumab-refractory, advanced or metastatic breast cancer. Drugs. 69(15): 2125-48.

Frank DA. (2009) Targeting transcription factors for cancer therapy. IDrugs 12(1): 29-33.

Franke, L. et al. (2006) Reconstruction of a functional human gene network, with an application for prioritizing positional candidate genes. Am J Hum Genet. 78(6):1011-25.

Freeman, L. C. (1977) A set of measures of centrality based on betweenness. *Sociometry* 40: 35–41.

Freeman, L. C. (1979) Centrality in social networks: conceptual clarification. *Soc. Networks* 1: 215-239.

Friedrich, M. et al. (2002) Analysis of vitamin D-receptor (VDR) and retinoid X-receptor alpha in breast cancer. Histochem J. 34: 35-40.

Fujii, T. et al. (2008) Preclinical and clinical studies of novel breast cancer drugs targeting molecules involved in protein kinase C signaling, the putative metastasis-suppressor gene Cap43 and the Y-box binding protein-1. Curr Med Chem. 15(6): 528-37.

Gary, D. et al. (2003) BIND: the Biomolecular Interaction Network Database. *Nucleic Acids Res.* 31(1): 248–50.

Genome News Network (<http://www.genomenewsnetwork.org/>), Oct. 2009.

Giantonio, B. J. et al. (2007) Bevacizumab in combination with oxaliplatin, fluorouracil, and leucovorin (FOLFOX4) for previously treated metastatic colorectal cancer: results from the Eastern Cooperative Oncology Group Study E3200. J Clin Oncol. 25(12): 1539-44.

Gilsdorf, M. et al. (2009) GenomeRNAi: a database for cell-based RNAi phenotypes. 2009 update. Nucleic Acids Res. 38(Database issue): D448-52.

Gligorov, J. and Lotz, J.P. (2008) Optimal treatment strategies in postmenopausal women with hormone-receptor-positive and HER2-negative metastatic breast cancer. Breast Cancer Res Treat. 112 Suppl 1:53-66.

Goff, L. W. et al. (2008) A phase I trial of irinotecan alternating with epirubicin in patients with advanced malignancies. Am J Clin Oncol. 31(5): 413-6.

Hanna, N. et al. (2006) Phase II trial of cetuximab in patients with previously treated non-small-cell lung cancer. J Clin Oncol. 24:5253–8.

Haura, E. B. et al. (2010) Phase I/II study of the Src inhibitor dasatinib in combination with erlotinib in advanced non-small-cell lung cancer. J Clin Oncol. 28(8): 1387-94.

Herold, C.I. et al. (2011) Phase II Trial of Dasatinib in Patients with Metastatic Breast Cancer Using Real-Time Pharmacodynamic Tissue Biomarkers of Src Inhibition to Escalate Dosing. Clin Cancer Res.[Epub ahead of print]

Hodge,A.E. et al. (2007) The PharmGKB: integration, aggregation, and annotation of pharmacogenomic data and knowledge. Clin Pharmacol Ther. 81(1): 21-4.

Hotte1, S. J. et al. (2006) Phase I trial of UCN-01 in combination with topotecan in patients with advanced solid cancers: a Princess Margaret Hospital Phase II Consortium study. Annals of Oncology 17(2): 334-340.

Hawkins, W. et al. (2005) Transient exposure of mammary tumors to PD184352 and UCN-01 causes tumor cell death in vivo and prolonged suppression of tumor regrowth. Cancer Biol Ther. 4(11): 1275-84.

Higgins, M. E. et al. (2006) CancerGenes: a gene selection resource for cancer genome projects. Nucleic Acids Res. 35(Database issue): D721-6.

Hopkins, A. L. and Groom, C. R. (2002) The druggable genome. Nat Rev Drug Discov. 1(9): 727-30.

Hurwitz, H. et al. (2004) Bevacizumab plus irinotecan, fluorouracil, and leucovorin for metastatic colorectal cancer. N Engl J Med. 350(23): 2335-42.

Iorns, E. et al. (2007) Utilizing RNA interference to enhance cancer drug discovery. Nat Rev Drug Discov. 6(7): 556-68.

Iorns, E. et al. (2009) Parallel RNAi and compound screens identify the PDK1 pathway as a target for tamoxifen sensitization. *Biochem J.* 417(1): 361-70.

Irizarry, R. A. et al. (2003) Exploration, normalization and summaries of high density oligonucleotide array probe level data. Biostatistics 4(2): 249-264.

Ji, D. et al. (2007) A screen of shRNAs targeting tumor suppressor genes to identify factors involved in A549 paclitaxel sensitivity. *Oncol Rep*. 18(6): 1499-505.

Jin, H. O. et al. (2008) A combination of sulindac and arsenic trioxide synergistically induces apoptosis in human lung cancer H1299 cells via c-Jun NH2-terminal kinase-dependent Bcl-xL phosphorylation. Lung Cancer 61(3): 317-27.

Johnson, D. H. et al. (2004) Randomized phase II trial comparing bevacizumab plus carboplatin and paclitaxel with carboplatin and paclitaxel alone in previously untreated locally advanced or metastatic non-small-cell lung cancer. J Clin Oncol 22: 2184-91.

Johnston, S. et al. (2009) Lapatinib combined with letrozole versus letrozole and placebo as first-line therapy for postmenopausal hormone receptor-positive metastatic breast cancer. J Clin Oncol. 27(33):5538-46.

Jones, S. et al. (2008) Core signaling pathways in human pancreatic cancers revealed by global genomic analyses. Science 321(5897): 1801-6.

Kanehisa, M., et al. (2010) KEGG for representation and analysis of molecular networks involving diseases and drugs. *Nucleic Acids Res.* 38: D355-D360.

Kaufman, B. et al. (2009) Trastuzumab plus anastrozole versus anastrozole alone for the treatment of postmenopausal women with human epidermal growth factor receptor 2-positive, hormone receptor-positive metastatic breast cancer: results from the randomized phase III TAnDEM study. J Clin Oncol. 27(33): 5529-37.

Keshava Prasad, T. S. et al. (2009) Human Protein Reference Database-2009 update. *Nucleic Acids Res.* 37(Database issue): D767-72.

Kitadai, Y. et al. (2006) Targeting the expression of platelet-derived growth factor receptor by reactive stroma inhibits growth and metastasis of human colon carcinoma. *Am J Pathol*. 169(6): 2054-65.

Koh, J. et al. (2002) UCN-01 (7-hydroxystaurosporine) inhibits the growth of human breast cancer xenografts through disruption of signal transduction. Breast Cancer. 9(1): 50-4.

Kohfeld, S. et al. (2007) 1-Aryl-4,6-dihydropyrazolo[4,3-d][1]benzazepin-5(1H)-ones: a new class of antiproliferative agents with selectivity for human leukemia and breast cancer cell lines. Eur J Med Chem. 42(11-12): 1317-24.

Köhler, S., Bauer, S., Horn, D., Robinson, P. N. (2008) Walking the interactome for prioritization of candidate disease genes. Am J Hum Genet. 82(4): 949-58.

Kopetz, S. et al. (2009) Synergistic activity of the SRC family kinase inhibitor dasatinib and oxaliplatin in colon carcinoma cells is mediated by oxidative stress. Cancer Res. 69(9):3842-9.

Köstner K (2009) The relevance of vitamin D receptor (VDR) gene polymorphisms for cancer: a review of the literature. Anticancer Res. 29(9):3511-36.

Knox,C. et al. (2011) DrugBank 3.0: a comprehensive resource for 'omics' research on drugs. Nucleic Acids Res. 39(Database issue): D1035-41.

Krause, D. S. and Van Etten, R. A. (2005) Tyrosine kinases as targets for cancer therapy. N Engl J Med. 353(2): 172-87.

Lane, M. E. et al. (2001) A novel cdk2-selective inhibitor, SU9516, induces apoptosis in colon carcinoma cells. Cancer Res. 61(16): 6170-7.

Lee, S. K. and Kumar, P. (2009) Conditional RNAi: towards a silent gene therapy. Adv Drug Deliv Rev. 61(7-8): 650-64.

Li, X. et al. (2004) Arsenic trioxide causes redistribution of cell cycle, caspase activation, and GADD expression in human colonic, breast, and pancreatic cancer cells. Cancer Invest. 22(3): 389-400.

Li, L. et al. (2006) A Framework of Integrating Gene Relations from Heterogeneous Data Sources: An Experiment on Arabidopsis Thaliana. *Bioinformatics* 22 (16): 2037-43.

Li, J. et al. (2010) A chemical and phosphoproteomic characterization of dasatinib action in lung cancer. Nat Chem Biol. 6(4): 291-9.

Liu, H. et al. (2003) Citron kinase is a cell cycle-dependent, nuclear protein required for G2/M transition of hepatocytes. J Biol Chem. 278(4): 2541-8.

Lin, E. et al. (2009) Exon array profiling detects EML4-ALK fusion in breast, colorectal, and non-small cell lung cancers. Mol Cancer Res 7(9): 1466-76.

Linding, R. et al. (2007) Systematic discovery of in vivo phosphorylation networks. *Cell* 129(7): 1415-1426.

Linding, R. et al. (2008) NetworKIN: a resource for exploring cellular phosphorylation networks. *Nucleic Acids Res.* 36(Database issue): D695–9.

Linghu, B. et al. (2009) Genome-wide prioritization of disease genes and identification of disease-disease associations from an integrated human functional linkage network. Genome Biol. 10(9): R91.

Luo, B. et al. (2008) Highly parallel identification of essential genes in cancer cells. *Proc Natl Acad Sci.* 105(51): 20380-5.

Luo, J. et al. (2009) A genome-wide RNAi screen identifies multiple synthetic lethal interactions with the Ras oncogene. Cell 137(5): 835-48.

Luu, T. H. et al. (2008) A phase II trial of vorinostat (suberoylanilide hydroxamic acid) in metastatic breast cancer: a California Cancer Consortium study. Clin Cancer Res. 14(21): 7138-42.

Mills, E. et al. (2005) Melatonin in the treatment of cancer: a systematic review of randomized controlled trials and meta-analysis. J Pineal Res. 39(4):360-6.

Mueller, L. et al. (2007) Imatinib mesylate inhibits proliferation and modulates cytokine expression of human cancer-associated stromal fibroblasts from colorectal metastases. Cancer Lett. 250(2):329-38.

Meyerson, M. et al. (2010) Advances in understanding cancer genomes through second-generation sequencing. Nature Reviews Genetics 11, 685-696

Moffat, J. et al. (2006) A lentiviral RNAi library for human and mouse genes applied to an arrayed viral high-content screen. *Cell* 124(6): 1283-98.

Navara, K. J. and Nelson, R. J. (2007) The dark side of light at night: physiological, epidemiological, and ecological consequences. J Pineal Res. 43(3):215-24.

Newcomb, E. W. (2004) Flavopiridol: pleiotropic biological effects enhance its anti-cancer activity. Anticancer Drugs. 15(5): 411-9.

Newman, M. E. J. (2003) The structure and function of complex networks. SIAM Rev. 45: 167.

Newman, M. E. J. (2004) Analysis of weighted networks. Phys. Rev. E 70(5): 1-9.

Ng S. K. et al. (2003) InterDom: a database of putative interacting protein domains for validating predicted protein interactions and complexes. *Nucleic Acids Research* 31(1): 251-4.

Obayashi T. et al. (2008) COXPRESdb: a database of coexpressed gene networks in mammals. *Nucleic Acids Res.* 36(Database issue): D77-82.

Pal, S. K. et al. (2010) Targeted therapies for non-small cell lung cancer: an evolving landscape. Mol Cancer Ther. 9(7): 1931-44.

Perotti, C. et al. (2008) Heat shock protein-90-alpha, a prolactin-STAT5 target gene identified in breast cancer cells, is involved in apoptosis regulation. Breast Cancer Res. 10(6): R94.

Polyak, K. et al. (1997) A model for p53-induced apoptosis. Nature 389(6648): 300-5.

Poynter, J. N. et al. (2005) Statins and the risk of colorectal cancer. N Engl J Med. 352(21):2184-92.

Qiu, J. and Noble, W. S. (2008) Predicting co-complexed protein pairs from heterogeneous data. *PLoS Computational Biology* 4(4):e1000054.

Radhakrishnan, Y. et al. (2008) Insulin-like growth factor-I stimulates Shc-dependent phosphatidylinositol 3-kinase activation via Grb2-associated p85 in vascular smooth muscle cells. J Biol Chem. 283(24): 16320-31.

Reck M. et al. (2009) Phase III trial of cisplatin plus gemcitabine with either placebo or bevacizumab as first-line therapy for nonsquamous non-small-cell lung cancer: AVAiL. J Clin Oncol 27: 1227–34.

Rhodes, D. R., et al. (2005) Probabilistic model of the human protein-protein interaction network. *Nat. Biotechnol.* 23: 951–9.

Russ, A. P. and Lampel, S. (2005) The druggable genome: an update. Drug Discov Today. 10(23-24):1607-10.

Saar-Tsechansky, M. and Provost, F. (2007) Handling missing values when applying classification models. Journal of Machine Learning Research 8: 1625-57.

Sawyers C. (2004) Targeted cancer therapy. Nature 432(7015):294-7.

Scagliotti, G. et al. (2010) Phase III study of carboplatin and paclitaxel alone or with sorafenib in advanced non-small-cell lung cancer. J Clin Oncol. 28(11): 1835-42.

Schapire, R. E. and Singer, Y. (1999) Improved boosting algorithms using confidence-rated predictions. Machine Learning 37(3): 297–336.

Schlabach, M. R. et al. (2008) Cancer proliferation gene discovery through functional genomics. *Science* 319(5863): 620-4.

Schonn, I. et al. (2009) Cellular responses to etoposide: cell death despite cell cycle arrest and repair of DNA damage. Apoptosis. 2009.

Seet, B.T. et al. (2006) Reading protein modifications with interaction domains. Nat Rev Mol Cell Biol. 7(7): 473-83.

Seng, S. et al. (2007) The nuclear matrix protein, NRP/B, enhances Nrf2-mediated oxidative stress responses in breast cancer cells. Cancer Res. 67(18): 8596-604.

Seng, S. et al. (2009) NRP/B mutations impair Nrf2-dependent NQO1 induction in human primary brain tumors. Oncogene. 28(3): 378-89.

Shawver, L. K. et al. (2002) Smart drugs: tyrosine kinase inhibitors in cancer therapy. Cancer Cell 1(2): 117-23.

Silva J. M. et al. (2008) Profiling essential genes in human mammary cells by multiplex RNAi screening. *Science* 319(5863): 617-20.

Simpson, K. J. et al. (2008) Identification of genes that regulate epithelial cell migration using an siRNA screening approach. *Nat Cell Biol.* 10(9): 1027-38.

Sjöblom, T et al. (2006) The consensus coding sequences of human breast and colorectal cancers. Science, 314(5797): 268-74.

Socinski, M. A. et al. (2008) Multicenter, Phase II trial of sunitinib in previously treated, advanced non-small-cell lung cancer. J Clin Oncol 26: 650–6.

Swanton, C. et al. (2007) Regulators of mitotic arrest and ceramide metabolism are determinants of sensitivity to paclitaxel and other chemotherapeutic drugs. *Cancer Cell* 11(6): 498-512.

Taher, T. E. et al. (2002) c-Cbl is involved in Met signaling in B cells and mediates hepatocyte growth factor-induced receptor ubiquitination. J Immunol.169(7): 3793-800.

Takagi, K. et al. (2008) CDK inhibitor enhances the sensitivity to 5-fluorouracil in colorectal cancer cells. Int J Oncol. 32(5): 1105-10.

Tao, M. et al. (2009) ITCH K63-ubiquitinates the NOD2 binding protein, RIP2, to influence inflammatory signaling pathways. Curr Biol. 19(15): 1255-63.

Tavazoie, S. et al. (1999) Systematic determination of genetic network architecture. *Nature Genetics* 22(3): 281-5.

Taylor, I. W. et al. (2009) Dynamic modularity in protein interaction networks predicts breast cancer outcome. Nat Biotechnol. 27(2): 199-204.

The Cancer Genome Atlas Research Network (TCGA) (2008) Comprehensive genom-ic characterization defines human glioblastoma genes and core pathways, Nature 455(7216): 1061-8.

Tipping, M.E. (2001) Sparse Bayesian learning and the Relevance Vector Machine. Journal of Machine Learning Research 1: 211-44.

Turner, N. C. et al. (2008) A synthetic lethal siRNA screen identifying genes mediating sensitivity to a PARP inhibitor. *EMBO J.* 27(9): 1368-77.

Vastrik, I. et al. (2007) Reactome: a knowledge base of biologic pathways and processes. *Genome Biology* 8: R39.

Walker, T. et al. (2009) Sorafenib and vorinostat kill colon cancer cells by CD95-dependent and -independent mechanisms. *Mol Pharmacol*. 76(2): 342-55.

Wang, Y. et al. (2009) Effect of staurosporine on the mobility and invasiveness of lung adenocarcinoma A549 cells: an in vitro study. BMC Cancer 9: 174.

Wardley, A. M. et al. (2009) Randomized Phase II Trial of First-Line Trastuzumab Plus Docetaxel and Capecitabine Compared With Trastuzumab Plus Docetaxel in HER2-Positive Metastatic Breast Cancer. J Clin Oncol. 2009 Dec 28.

Wesierska-Gadek, J. et al. (2004) Cell cycle arrest induced in human breast cancer cells by cyclin-dependent kinase inhibitors: a comparison of the effects exerted by roscovitine and olomoucine. Pol J Pharmacol. 56(5): 635-41.

Wishart, D.S. et al. (2008) DrugBank: a knowledgebase for drugs, drug actions and drug targets. Nucleic Acids Res. 36(Database issue): D901-6.

Witters, L. M. et al. (2004) Combining flavopiridol with various signal transduction inhibitors. Oncol Rep. 11(3): 693-8.

Wood, L. D. et al. (2007) The genomic landscapes of human breast and colorectal cancers. Science 318(5853): 1108-13.

Wu, C. C., Asgharzadeh, S., Triche, T. J., and D'Argenio, D. Z. (2010) Prediction of Human Functional Genetic Networks from Heterogeneous Data Using RVM-Based Ensemble Learning. *Bioinformatics* 26(6): 807-13.

Xi, L. et al. (2008) Whole genome exon arrays identify differential expression of alternatively spliced, cancer-related genes in lung cancer. Nucleic Acids Res. 236(20): 6535-47.

Yang, C. Y., et al. (2008) PhosphoPOINT: a comprehensive human kinase interactome and phospho-protein database. *Bioinformatics* 24(16): i14-i20.

Yang, Z. et al. (2010) Synergistic actions of atorvastatin with gamma-tocotrienol and celecoxib against human colon cancer HT29 and HCT116 cells. *Int J Cancer.* 126(4): 852-63.

Ye, J. et al. (2005) Inhibition of mitogen-activated protein kinase kinase enhances apoptosis induced by arsenic trioxide in human breast cancer MCF-7 cells. Clin Exp Pharmacol Physiol. 32(12): 1042-8.

Yu, H. et al. (2007) The importance of bottlenecks in protein networks: correlation with gene essentiality and expression dynamics, PLoS Comput Biol. 3(4): e59.

Zhang, L. et al. (2007) Integrative genomic analysis of phosphatidylinositol 3'-kinase family identifies PIK3R3 as a potential therapeutic target in epithelial ovarian cancer. Clin Cancer Res. 13(18 Pt 1): 5314-21.

Zhang W et al. (2007) FCGR2A and FCGR3A polymorphisms associated with clinical outcome of epidermal growth factor receptor expressing metastatic colorectal cancer patients treated with single-agent cetuximab. *J Clin Oncol*. 25(24): 3712-8.

Zhu,F. et al. (2009) Update of TTD: Therapeutic Target Database. Nucleic Acids Res. 38(Database issue): D787-91.
